# The future of public health policymaking after COVID-19: a qualitative systematic review of lessons from Health in All Policies

**DOI:** 10.12688/openreseurope.13178.2

**Published:** 2021-07-21

**Authors:** Paul Cairney, Emily St Denny, Heather Mitchell

**Affiliations:** 1 History, Heritage, and Politics, University of Stirling, Stirling, FK94LA, UK; 2 Department of Political Science, University of Copenhagen, Copenhagen, DK-1353, Denmark; 3 Faculty of Health Sciences, University of Stirling, Stirling, FK94LA, UK

**Keywords:** Policymaking, Policy theory, Health in All Policies, Power, Complexity, Practical lessons, Health equity, COVID-19

## Abstract

**Background**: ‘Health in All Policies’ (HiAP) describes the pursuit of health equity. It has five main elements: treat health as a human right; identify evidence of the ‘social determinants’ of health inequalities, recognise that most powers to affect health are not held by health departments, promote intersectoral policymaking and collaboration inside and outside of government, and generate political will. Studies describe its potential but bemoan a major implementation gap. Some HiAP scholars learn from policymaking research how to understand this gap, but the use of policy theories is patchy. In that context, our guiding research question is: *How does HiAP research use policy theory to understand policymaking?* It allows us to zoom-out to survey the field and zoom-in to identify: the assumed and actual causes of policy change, and transferable lessons to HiAP scholars and advocates.

**Methods:** Our qualitative systematic review (two phases, 2018 and 2020) identified 4972 HiAP articles. Of these, 113 journal articles (research and commentary) provide a non-trivial reference to policymaking (at least one reference to a policymaking concept). We use the 113 articles to produce a general HiAP narrative and explore how the relatively theory-informed articles enhance it.

**Results**: Most articles focus on policy analysis (identifying policy problems and solutions) rather than policy theory (explaining policymaking dynamics). They report a disappointing gap between HiAP expectations and policy outcomes. Theory-informed articles contribute to a HiAP playbook to close that gap or a programme theory to design and evaluate HiAP in new ways.

**Conclusions**: Few HiAP articles use policy theories for their intended purpose. Policy theories provide lessons to aid critical reflection on power, political dilemmas, and policymaking context. HiAP scholars seek more instrumental lessons, potentially at the cost of effective advocacy and research.

## Plain language summary

Coronavirus disease 2019 (COVID-19) should have prompted governments to treat population health improvement as fundamental to public policy. Many made strong commitments to strategies to prevent an epidemic of non-communicable diseases (NCDs). They address the ‘social determinants’ of health, defined by the World Health Organization ( WHO, 2020a) as ‘the unfair and avoidable differences in health status … shaped by the distribution of money, power and resources’ and ‘the conditions in which people are born, grow, live, work and age’. Health in All Policies (HiAP) is the main vehicle, underpinned by: a commitment to health equity by addressing social determinants; the recognition that most health policies are not controlled by health departments; the need for collaboration across (and outside) government; and, the search for political commitment.

COVID-19 reinforces this rationale, highlighting the social determinants of social distancing and the mortality of people with NCDs. Yet, health departments postponed health improvement and moved resources to health protection. This experience challenges the assumption that the logic of health improvement is irresistible. Instead, HiAP momentum can be lost at any time.

In that context, we need more realistic lessons for public health. To that end, this review identifies lessons from studies of HiAP and policymaking. It suggests that HiAP advocates produced a 7-point playbook for the wrong game, contributing to a major gap between HiAP commitment and outcomes. Some describe the need to use policy research to produce new ways to promote and evaluate HiAP, but most do not use it effectively. 

We show how policymaking research helps to explain and understand the meaning of a HiAP implementation gap. Its main lesson is that policy outcomes are beyond the control of policymakers and HiAP advocates. As such, its practical lessons come from critical reflection on power and politics, not the reinvention of a playbook.

## Introduction

Health in All Policies (HiAP) scholars need to understand the policymaking processes that constrain or facilitate public health policy. Yet, very few HiAP studies are informed meaningfully by policymaking research. Consequently, research (a) tells an incomplete story of limited policy progress, which (b) exacerbates the problem it describes. To demonstrate, we describe the low status and progress of health improvement, how HiAP studies try to explain it, and how a greater focus on policy process research would help.

Coronavirus disease 2019 (COVID-19) has added an ironic twist to the low status of population health improvement policy. Health protection and improvement are symbiotic, and COVID-19 should have prompted governments across the world to treat health improvement as central to public policy. There were two main reasons to expect this symbiotic relationship. First, governments and international organisations had already made a strong rhetorical commitment to two elements of public health strategies:

1. *health protection,* to inoculate whole populations against epidemics or pandemics of communicable diseases; and,
2. *health improvement*, to prevent epidemics of non-communicable diseases (NCDs) such as heart disease, strokes, cancers, and diabetes.

The global commitment to health improvement is summed up by HiAP, based on researching the ‘social determinants’ of health and pursuing health equity via policymaking reforms. The World Health Organisation ( WHO, 2020a) describes ‘the unfair and avoidable differences in health status’ that are ‘shaped by the distribution of money, power and resources’ and ‘the conditions in which people are born, grow, live, work and age’. Its work is based on the argument that health is a human right, and that a population’s health inequalities are unfair and caused by differences in income, occupation, education, and living conditions. This commitment to health equity connects to a narrative of policymaking: the most useful health policies are not controlled by health sectors, and success requires intersectoral action and collaboration inside and outside government, built on high-level political commitment.

Second, COVID-19 reinforces the importance of the social determinants of health and the need for intersectoral action. Many inequalities relating to income, housing, and social and environmental conditions cause inequalities in the distribution of NCDs *and* people’s ability to protect themselves from infectious disease ( Marmot *et al.*, 2020). COVID-19 had a visibly disproportionate impact on people with (a) underlying health conditions associated with NCDs (which increased the risk of death or major illness), *and* (b) less ability to live and work safely (while social distancing). Further, the level of global and domestic action to address COVID-19 shows what is possible with enough political commitment.

Instead, it helped side-line improvement, reinforcing the sense that it enjoys high rhetorical support but low follow-through. Most health departments and agencies postponed health improvement strategies and moved resources to health protection ( WHO, 2020b).

This experience is familiar to researchers and advocates of health improvement. It reinforces two key messages. First, the evidence does not speak for itself. The cumulative weight of research evidence on social determinants is clear to public health specialists, who lament the large gap between the size of the policy problem and the response of governments. The idea of social determinants is less well known or convincing to policymakers or most publics. Second, the logic of health improvement is not irresistible. HiAP advocates tell a remarkably similar story of what should be done, but it is not widely understood.

These conclusions are true even when government strategies express high rhetorical commitment to evidence-based and preventive public health. A government’s commitment does not lead inevitably to the delivery of a fully-formed HiAP model. Defining a policy problem does not initiate an inevitable series of policymaking ‘stages’ such as selecting evidence-based solutions to be implemented and evaluated. There is always a major gap between the idea of HiAP and its implementation, it is difficult to generate HiAP momentum for the long term, and it can be lost at any time. Indeed, the main finding from the study of HiAP is an implementation gap caused by low ‘ownership’ of health improvement and insufficient ‘political will’ to protect it.

These experiences suggest that we need to generate more realistic lessons for health improvement from policy process research. Policy theories guide empirical research by showing how specific policy agendas relate to a general policymaking context. However, most HiAP research does not appear to draw on these lessons, and most rely on outmoded conceptions of policymaking (such as the ‘policy cycle’). Further, most theory-informed HiAP research focuses on policy analysis: the identification of policy problems and solutions. It combines a *functional logic* (what we need to happen) with *programme logic* (what we think we need to do to achieve it) and uses theories to provide *practical lessons* to that end. Such analysis is based more on hope than reality and remains incomplete without the intensive study of policymaking. To that end, we identify lessons from studies of HiAP *and* policymaking.

## Methods

Our guiding question is: *How does Health in All Policies research use policy theory to understand policymaking?* Originally, we identified five sub-questions:

1. How many studies of HiAP provide a non-trivial reference to policymaking concepts or theories?
2. How do these HiAP studies describe policymaking?
3. How do these studies describe the ‘mechanisms’ of policy change (in other words, the causes of policy change that are vital to HiAP strategies)?
4. What transferable lessons do studies of HiAP provide? For example, what lessons for other governments do HiAP case studies provide?
5. How do HiAP studies relate health equity to concepts such as spatial justice?
  (see PROSPERO record and PRISMA checklist; we answer question 5 in Cairney *et al.*, 2022 for the Horizon2020 project IMAJINE).

We refined our search strategy and inclusion criteria after learning from comparable research and research-in-progress, but adapted in two ways.

First, to identify the use of policy theory in HiAP studies, we follow Embrett & Randall (2014) on its use in ‘Social determinants of health and health equity policy research’, and Munro & Cairney (2020) on energy systems research. However, we initially set a lower bar for inclusion than those studies, then read the full text to identify theory-informed discussions of policymaking. We present three reasons for this approach:

1. Our previous work suggests that researchers generally describe their expectations for policymaking reform without citing studies of policymaking ( Cairney, 2016; Cairney & St Denny, 2020).
2. Previous reviews suggest that this wide search parameter does not produce an unmanageable number of articles ( Embrett & Randall, 2014; Munro & Cairney, 2020).
3. High initial inclusion (113 articles, up to June 2020) helps to identify a common HiAP narrative based on a well-established policy agenda but insufficient reference to policy studies. We compare this general narrative to more theory-informed HiAP studies.

Second, we learned from the protocol by Such *et al.* (2019) on how to research the *dynamics* of HiAP-inspired intersectoral collaboration (we then modified our own Prospero record on 17.6.21 to address a too-close reliance on Such *et al*.’s original wording). We had also planned to focus on the *mechanisms* of HiAP (inspired by Pawson *et al.*’s, 2005 agenda on realist review). However, it became clear that too few articles provided enough information to proceed. Most present a too-vague discussion of policymaking causality, making it impractical to answer the question ‘What works for whom in what circumstances and in what respects?’ (2005: 12). Some describe mechanisms and causality in relation to programme logic ( Lawless *et al.*, 2018).

We searched eight databases (Web of Science, Applied Social Science Index and Abstracts [ASSIA], Centre for Reviews and Dissemination [CRD], the Cochrane Library, Scopus, ProQuest, TRIP, and PROSPERO) in 2018 (search ran 28 May to 25 June) and 2020 (search 22 June to 29 July), using the same protocol ( Table 1 and Table 2). We used these search terms: (1) HiAP, Health in All Polic* (and Healthy Public Polic* and ‘integrated health polic*’ to include articles written before HiAP’s routine use); combined with (2) articles providing one or more references to (a) the ‘policy cycle’ (or a particular stage, such as agenda setting or implementation) or (b) an established policy theory (such as multiple streams, the advocacy coalition framework, punctuated equilibrium theory) or concept (such as forms of new institutionalism). Word limits prevent us from summarising these theories in this paper. Instead, we use Cairney, 2020a for a list of included theories and concepts (summarised on Cairney’s blog).

**Table 1.** Search 1 (May–June 2018).

Database	Search results	Duplicates	No access	Excluded	Included
Web of Science	409	53	13	133	210
ASSIA	92	0	0	92	0
CRD	2	0	0	2	0
Cochrane	8	1	0	4	3
Scopus	260	20	5	190	45
ProQuest	889	34	4	842	9
TRIP	87	17	0	68	2
PROSPERO	0	0	0	0	0
**Combined** **total**	**1747**	**125**	**22**	**1331**	**269**

**Table 2.** Search 2 (June–July 2020).

Database	Search results	Duplicates	No access	Excluded	Included
Web of Science	559	90	17	170	282
ASSIA	515	147	8	319	41
CRD	2	0	0	2	0
Cochrane	10	1	0	6	3
Scopus	467	187	8	219	53
ProQUEST	1515	154	8	1315	38
TRIP	150	33	1	106	10
PROSPERO	7	0	0	7	0
**Combined total**	**3225**	**612**	**42**	**2144**	**427**

We used the following additional criteria for inclusion. The article had to: be published in a peer reviewed journal (including research and commentary articles); describe policymaking (we excluded articles that only described HiAP); and provide at least one reference to a conceptual study of policymaking in its bibliography (including articles that cite a relevant HiAP article rather than the original source). We identify the following risks of bias among the selected studies. We did not restrict by geography, but most included articles come from Australia and Western Europe. We did exclude on the basis of language: the text had to be available in English. We did not exclude on the basis of quality, but half of included articles drew on new research (primarily from documentary analysis and semi-structured interviews of HiAP participants, with some conducting surveys and focus groups) while half provided (generally unsystematic) literature reviews or commentary (including peer-reviewed commentaries on *International Journal of Health Policy and Management* articles by Carey & Friel, 2015 and Lawless *et al.*, 2018). While several articles drew on, and enhanced, the field of *public administration*, zero articles were written for publication in a *public policy* journal.

**Figure 1.**
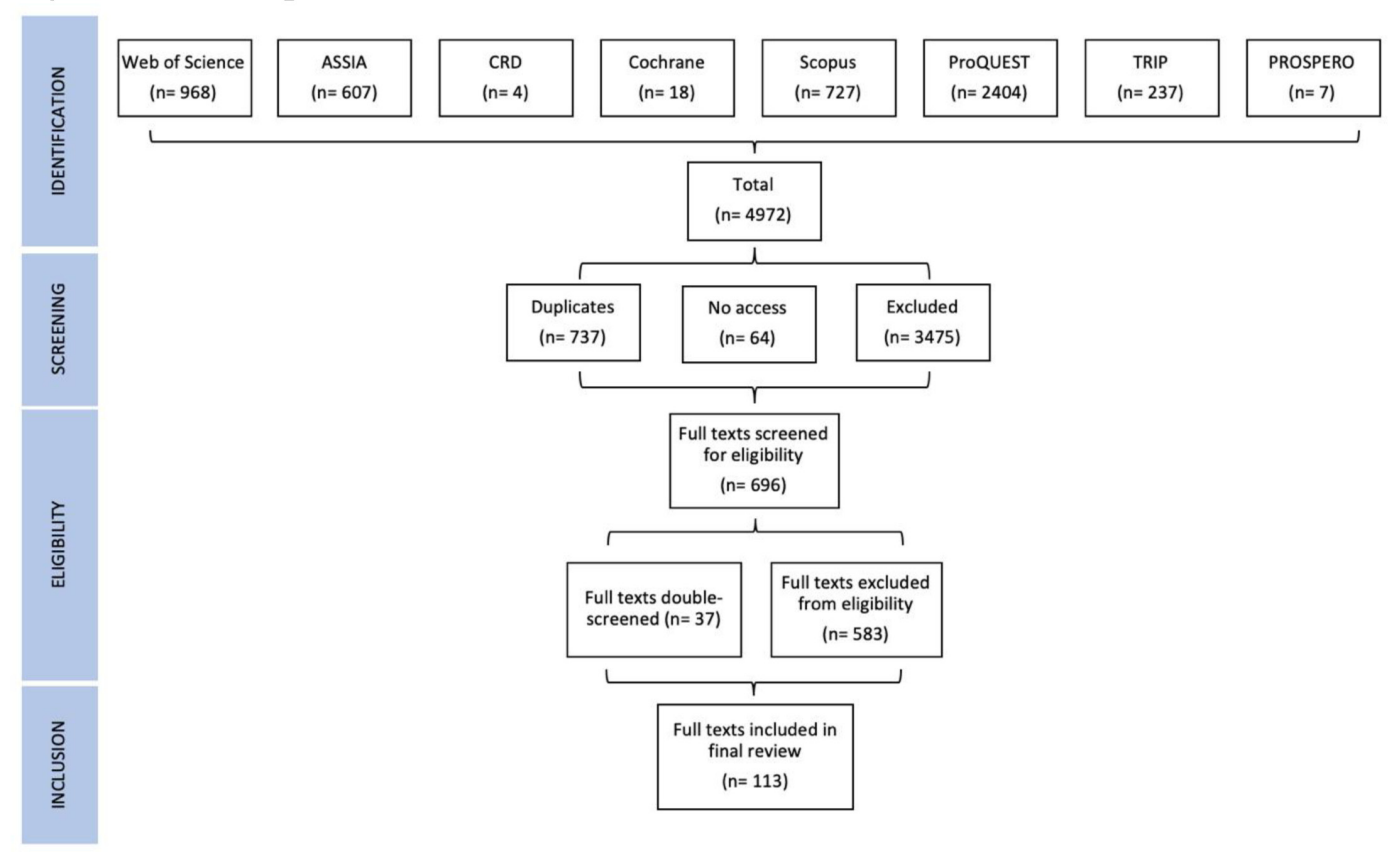
Review process flow-chart.

St Denny and Mitchell carried out double-screen spot-checks during the initial identification and screening phases, then Cairney and St Denny or Mitchell double screened 37 borderline cases during the final eligibility phase. In this stage, we excluded two borderline cases but included six that provided a comparable study of policymaking without citing ‘mainstream’ policy theories, such as by citing relevant authors (including Foucault) or concepts (such as systems approaches). In total, 113 articles were included in the final study ( Figure 1).

We extracted the following information from each article: the definition of HiAP and/or the context to justify the article’s focus, the ‘story’ of the paper (a summary of its key messages), what governments can learn, the role of politics and policymaking, country of author affiliation, country/region of study, policy sector or case study issue, the theory or concept discussed, the ‘stage’ of the ‘policy cycle’ discussed, methods, article type (e.g. research, review, commentary). We also extracted information to inform a sub-question: the role of ‘space’ or ‘territory’ in explanation ( Cairney *et al.*, 2022, for project IMAJINE). We sought in vain to extract systematically: *what works, for whom, in what respects, why*.

We used an inductive qualitative approach to identify key categories and themes from each paper. Articles generally focused on one or more of these elements:

1. A general narrative of HiAP as a way to address social determinants.
2. Advice for HiAP advocates based on a case study of experience (which we describe as the *HiAP playbook*).
3. Describing the experience of HiAP policymaking in a country or region.
4. Using policy theories to provide practical lessons for advocacy and evaluation.

## Results

### There is a commonly-told but vague HiAP narrative

There is a coherent but vague HiAP narrative in this set of articles ( St Denny *et al.*, 2021). Its key elements are:

*1. Treat health as a human right*

The *Helsinki Statement on Health in All Policies* (2014) is the outcome of the 8th Global Conference on Health Promotion (facilitated by the WHO) and a key reference point for HiAP studies. It describes health as a human right to be supported by governments and international organisations, treats health inequalities as ‘unfair and avoidable’ and exacerbated by commercial interests, introduces HiAP and describes it as essential to the UN Millennium Development Goals:

‘We call on governments to fulfil their obligations to their peoples’ health and well-being by taking the following actions: Commit to health and health equity as a political priority by adopting the principles of Health in All Policies and taking action on the social determinants of health’.

*2. Identify evidence of the ‘social determinants’ of health inequalities*

Health is unequally distributed, and the cause is social, economic, and political rather than biological or caused primarily by individual choices: ‘all systematic differences in health between different socioeconomic groups within a country’ are unfair and avoidable, since ‘there is no biological reason for their existence’ and ‘systematic differences in lifestyles between socioeconomic groups are to a large extent shaped by structural factors’ ( Solar & Urwin, 2010: 6; Whitehead & Dahlgren, 2006: 4). HiAP advocates seek to measure and address the:

‘significant and persistent disparities in health outcomes caused by structural inequities in social and economic factors, including employment opportunities, the law and the justice systems, education, housing, neighborhood environments, and transportation. These elements are otherwise known as the social determinants of heath. The opportunity or lack of opportunity to be healthy is too often associated with a person’s socioeconomic status, race, ethnicity, gender, religion, sexual identity, or disability’ ( Bliss *et al.*, 2016: S88).

*3. Identify evidence-based ‘upstream’ solutions*

Identify the policy instruments (‘interventions’) to improve the social and economic environment, supported by analytical tools - including health impact assessments (HIAs) – to monitor the impact of other policies on that environment. Public health approaches tend to emphasise one or more of three concepts.

First, select preventive approaches to health improvement, to intervene as early as possible in people’s lives, encouraging primary prevention (akin to whole population inoculations from infectious disease) more than secondary (targeting at-risk groups) or tertiary (mitigating the effects of a known condition in groups) ( Cairney & St Denny, 2020).

Second, focus on ‘upstream’ interventions. HiAP metaphors on systems or ecology highlight the shift from a focus on individual-centred interventions (downstream) towards the environments relevant to whole populations (upstream). The analogy helps challenge a dominant focus on healthcare and individual lifestyles ( Bharmal *et al.*, 2015; Williams *et al.*, 2008). HiAP accounts generally distinguish between: *upstream* measures to address whole populations and their socio-economic and physical environments; *midstream* measures to reduce the risk of harm to target populations, and *downstream* measures on ‘lifestyles’ or access to services:

‘Upstream interventions are aimed at fundamental social and economic reform and involve mechanisms for the redistribution of wealth, power, opportunities, decision‐making capacities, and other resources. Midstream interventions aim to reduce risky behaviors or exposures to hazards and may include strategies to affect lifestyle or psychosocial factors, and/or to improve material working and living conditions. Finally, downstream interventions aim to mitigate the inequitable impacts of upstream and midstream determinants of health and disease through efforts to increase equitable access to health care services’ ( Shankardass *et al.*, 2011: 29).
‘Upstream interventions involve policy approaches that have the potential to affect large populations through regulation, increasing access, or economic incentives. Midstream interventions occur within organizations, such as worksites. Downstream interventions involve individual-level behavioral approaches for prevention or disease management’ ( Brownson *et al.*, 2010: 6).

Third, identify the wider causal dynamics of health, with social determinants representing ‘causes of the causes of health’ and politics/policymaking representing the ‘causes of the causes of the causes’ ( De Leeuw & Peters, 2015: 987–8; Oni *et al.*, 2019).

These descriptions are broad, producing many ways to describe priorities, including:

- Improve ‘daily living conditions’, challenging the ‘inequitable distribution of power, money, and resources’, and establishing surveillance to measure inequalities and the impact of policies ( WHO Commission on the Social Determinants of Health)
- Encourage early interventions for children and ‘proportionate universalism’, using universal public services to provide more support based on ‘the level of disadvantage’ ( Marmot review of health inequalities in England, 2010).
- Facilitate access to high quality education and employment, improve housing and public transport, air, water, or food quality, and reduce domestic and community violence ( Gottlieb *et al.*, 2012; Storm *et al.*, 2011).
- Identify groups vulnerable to discrimination, such as when people of colour have less access to high quality housing, employment, education, or healthcare, and face more violence or government discrimination ( Corburn *et al.*, 2014: 627; Hall *et al.*, 2016).
- Address an ‘obesogenic environment’, using regulations, planning, and economic incentives to improve access to healthy food and safe places to exercise ( Alderwick & Gottlieb, 2019: 411; Kranzler *et al.*, 2013: 3).

*4. Promote intersectoral action and collaborative governance*

Most powers to affect population health are not held by health departments. Major responsibilities – to redistribute income, improve public services, reduce discrimination, and improve social, economic, and physical environments - are distributed across government departments (and multiple levels and types of government). Their implementation relies on cooperation by many non-governmental actors. Hence, intersectoral action and collaborative approaches to policymaking are central to HiAP, ‘from the inception to the end of policy development’ ( Harris *et al.*, 2012). The *Helsinki Statement* emphasises a need for ‘policy coherence’, fostered by ‘effective structures, processes and resources that enable implementation of the Health in All Policies approach across governments at all levels and between governments’.

Descriptions of this aim vary according to the political systems that determine the distribution of HiAP responsibilities. Further, we can detect differences in focus, such as on: ‘interdepartmental coordination’ or ‘joining up’ ( Carey & Friel, 2015: 796); ‘whole-of-government’ approaches that combine a HiAP strategy and unit, the routine use of HIAs, and collaboration inside and outside of government ( Storm *et al.*, 2014: 184); or a ‘dynamic policy response across portfolio boundaries by governance networks, consisting of governmental as well as societal actors’ ( Peters *et al.*, 2016: 290–1). They reflect the multiple ways to pursue HiAP via intersectoral action:

‘First, policy sectors other than health can be encouraged or explicitly asked to adopt policies that advance the health objectives. Second, policy integration can consist of launching specific policy measures that help to mutually attain the objectives of health policy and other policy sectors. Third, actors from the health sector can make their health expertise available to other policy sectors. This approach would mean that the health sector strives to promote health objectives through systematic cooperation with other policy sectors. Fourth, policy integration can be realised by assessing and possibly addressing the health effects of policy proposals from other policy sectors’ ( Tosun & Lang, 2017: 555).

*5. Seek high and enduring political commitment*

High level political support is crucial to the production of an ambitious strategy document and to dedicate resources to its implementation. Consistently high commitment from elected policymakers keeps HiAP on the policy agenda and helps cut through ‘administrative silos’ and address ‘turf wars’ ( Carey & Crammond, 2015). Or, low or fleeting commitment is the reason for poor implementation. 

Overall, we find a remarkable level of in-principle agreement on those elements. This consensus relates partly to the vagueness of HiAP ( Alderwick & Gottlieb, 2019), and a tendency for studies to use the same foundational sources (such as the *Helsinki Statement*). In practice, the meaning of ‘social determinants’ of health, ‘upstream’ measures, collaboration across and outside of government, ‘political will’, and auxiliary terms such as ‘communities’ and ‘boundaries’, are not well defined ( Baum *et al.*, 2020; Carey & Friel, 2015; de Leeuw, 2016; O’Flynn, 2016; Post *et al.*, 2010). This misplaced sense of HiAP coherence contributes to confusing advice on how to operationalise and deliver HiAP aims.

## The HiAP playbook

The WHO (2020c) ‘tobacco control playbook’ describes a ‘living document’, ‘developed by collecting numerous evidence-based arguments from different thematic areas, reflecting the challenges that tobacco control leaders have faced while implementing’ the *Framework Convention on Tobacco Control*. There is no direct HiAP equivalent (although see Lazzari *et al.*, 2015: 279 and Howard & Gunther, 2012 for ‘top tips for implementing HiAP’). Rather, we describe its playbook as the seven most common pieces of advice from HiAP research. They combine to produce a plausible but often-misleading strategy.

*1. Use well-established ways to get from talk to action, and to sustain long-term commitment*

The WHO (2014: 12–17) ‘starter kit’ has six components:

1. *Establish the need and priorities for HiAP*. Generate demand in relation to ‘gaps’ in health and services, and your political system’s context.
2. *Frame planned action*. Relate your HiAP strategy to what is feasible in each context.
3. *Identify supportive structures and processes*. Establish how the current leadership, governance, agendas, and norms of your system will aid HiAP.
4. *Facilitate assessment and engagement*. Promote tools to assess the health impacts of policy, relate them to target populations, and engage with policymakers to ensure this evidence is used for policy.
5. *Ensure monitoring, evaluation, and reporting*. Incorporate HiAP in the continuous process of policymaking, implementation, and evaluation.
6. *Build capacity*. Facilitate HiAP training and build capacity (staffing, research, guidance, experience) in health and non-health sectors and outside government.

Bauman *et al.* (2014b: i144) describe a 5-step approach to ensure that high commitment does not wane. First, define the policy problem in relation to the social and environmental determinants of health. Second, ‘build the case’ for action by identifying which sectors are responsible for each solution. Third, identify feasible action based on resources and political commitment. Fourth, produce a plan for policy implementation across multiple sectors. Finally, evaluate progress with an annual reporting system to foster accountability.

In each case, there is a balancing act between describing HiAP as a uniform model to be applied across the globe or a general approach with necessarily different outcomes. For example, the ‘starter kit’ should be adapted to reflect what is ‘relevant for their specific governance, economic and social contexts’, but is ‘applicable to all countries and policy contexts’ (2014: 8). In other words, a core group of HiAP advocates may share a common purpose and seek coherent approaches and policies each time, *and* recognise variation in the conduciveness of each context to HiAP, but there is little guidance on how to resolve these tensions.

*2. Raise awareness and connect HiAP to a government’s values and policy agendas*

Raise awareness of HiAP in other sectors, partly by framing HiAP aims to be consistent with a government’s overall vision and core business, in the hope that HiAP becomes mainstreamed throughout government policy and an accepted way to judge performance ( Greaves & Bialystock, 2011: 407). Examples include:

- ‘Understanding each sector’s needs and culture may be crucial to frame the need for HiAP in a way that places it on their agenda’ ( Freiler *et al.*, 2013: 1070)
- Describe ‘to policymakers why intersectoral action is needed to address population health and equity with the expectation that they will buy in and participate’, and convince ‘potential partners to adopt policies and measures that directly support health objectives’ ( Molnar *et al.*, 2016: 2–3).
- Social determinants may not get policymaker attention unless framed as a way to reduce the unsustainable burden on health services ( Kickbusch *et al.*, 2014: 187–8).
- ‘Promoting health will also assist in stimulating economic productivity and reduce the cost of health care’ ( Delany *et al.*, 2016: 888). This argument is essential if governments focus more on economic growth than health ( Harris *et al.*, 2012: 6).
- Health equity is essential to ‘EU core values such as solidarity, equity and universality’ ( Bert *et al.*, 2015: 45).
- Frame the use of HIAs in terms of meeting another sector’s objectives ( Shankardass *et al.*, 2015: 469).

However, studies also describe a tension between only encouraging preferred solutions (such as by emphasising their feasibility, Bowman *et al.*, 2012: 847) and ‘speaking truth to power’ to challenge dominant ways of thinking in government:

- Speak ‘hard truths’ about social determinants and health equity values, to make sure that policy meetings are not dominated by a focus on lifestyles ( Bliss *et al.*, 2016: S92).
- Describe (a) health’s wider socioeconomic and environmental determinants, and the need for ‘community action’ and cross-governmental cooperation, to (b) challenge a primary focus on individual lifestyle and health services and the sense that HiAP is solely a central government responsibility ( De Leeuw & Polman, 1995: 332).
- Provide a powerful narrative to challenge business-as-usual approaches ( Carey & Crammond, 2015: 1026).

Either way, HiAP studies describe the sense that awareness raising has a limited and fleeting impact. Breton (2016: 383–4) describe a tendency in government to return to a focus on healthcare and individual lifestyles (‘lifestyle drift’ - De Leeuw & Clavier, 2011: 237–40).

*3. Focus on win-win solutions to foster trust-based intersectoral action*

A key driver of intersectoral collaboration is a *win-win* or *mutual gains* approach. It demonstrates to non-health sectors that there can be a ‘shared vision across sectors’ ( Guglielmin *et al.*, 2018: 291), or that a health focus helps other sectors fulfil their own aims ( Freiler *et al.*, 2013). Producing specific, mutually beneficial, goals may foster collaboration and contribute to wider buy-in by fostering ‘cross-cultural understanding and mutual respect’ ( Bert *et al.*, 2015: 45; Kickbusch *et al.*, 2014: 187–92; Lawless *et al.*, 2018: 515; Newman *et al.*, 2016: 54). Molnar *et al.* (2016: 1; 8–12) describe trying to juggle many strategies, to:

- understand the aims and motivations of potential partners in other sectors
- signal the importance of ‘reciprocity’ to help each other deliver their own projects
- contribute to work on another sector’s agenda
- contribute to a ‘shared language’ to aid communication across sectors
- build on previously successful collaborations
- use high quality evidence of evaluations to show how a HiAP approach can reduce another sector’s costs or maximise efficiency
- use HIAs to foster coordination and ‘give credibility’ to policies developed in other policy sectors.

Van Eyk *et al.* (2020: 969) use the case study of child literacy programmes (South Australia) to show how to foster this approach. First, start ‘with the other sector’s business’ and be ‘responsive to the other sector’s professional culture and institutional logic’. Second, foster HiAP leadership and encourage policy champions to exploit opportunities for collaboration, focusing on helping other sectors understand the social determinants of health. Third, it helps when people in one sector have experience of another.

These approaches connect strongly to the recognition that trust is essential to collaboration inside and outside of government ( Cairney & Wellstead, 2020). Delany-Crowe *et al.* (2019; 178) follow Giddens to define trust in relation to:

‘Faith or confidence in another person, system or outcome; Calculations of reliability, or expectations of competence and/or goodwill; A belief in the likelihood of benefit stemming from relationships despite unknowns; A willingness to proceed despite the risk presented by the unknown’ (2019: 178).

They identify the role of trust in HiAP progress:

1. How to *create* trust to support joined-up government relationships. Factors include: the competence and skills of individuals, shared beliefs about the benefits of investment in HiAP, the confidence inspired by beneficial previous relationships and past experiences (2019: 179–82).
2. How to *maintain* trust. Foster reciprocity, regular communication, and shared ownership (2019: 182–3).
3. How trust can be *lost*. Trust diminishes (or distrust increases) when people feel let down by previous experiences. Examples include: vivid experiences when meetings took place after some knew the service would be cut; a sense of tokenism in some meetings; overly bureaucratic approaches; and, a failure to honour commitments. These factors exacerbate scepticism about the value of investing in HiAP (2019: 183–4).

There are two caveats. First, *the evidence of success is limited*. Kokkinen *et al.* (2019b) argue that, until very recently, almost no HiAP articles have provided evidence of the effectiveness of each strategy. Their comparative study (in California, Ecuador, Finland, Norway, Scotland and Thailand) highlights strong evidence for the effectiveness of: (1) developing a shared language for participants, and (2) fostering multiple strategic outcomes to incorporate the aims of many participants. They find less strong evidence for using public health arguments to bolster the case for policy change in other sectors (2019b: 7).

Second, *win-win approaches can have unintended consequences*. Holt *et al.* (2017: 881–2) suggest that intersectoral policymaking can ‘corrupt’ a social determinants approach (even in ‘best case’ countries such as Denmark, below). They found high cross-sectoral commitment, but also health managers struggling to frame intersectoral work in relation to the causes-of-the-causes and a tendency to focus on ‘downstream’ measures in other sectors (2017: 884).

*4. Avoid projecting a sense of ‘health imperialism’*

Avoid the sense that HiAP represents interference in non-health sectors ( Breton, 2016: 383–4), ‘where problems and the necessary actions are defined from the viewpoint of the health sector only’ ( Delany *et al.*, 2016: 889). This perception can:

- undermine collaboration ( Gottlieb *et al.*, 2012: 158; Lawless *et al.*, 2012: S15)
- exacerbate conflicts over jurisdiction ( Oneka *et al.*, 2017: 836)
- project the sense that the health sector is distributing extra work that will distract other sectors from their core business ( Guglielmin *et al.*, 2018: 287–90; Newman *et al.*, 2016: 54)
- trigger professional identity-based reactions against public health interference, exacerbated by low HiAP advocate respect for past achievements ( Synnevåg *et al.*, 2019: 7)

The HiAP ‘brand’ may be initially useful, to establish legitimacy for a focus on social determinants, intersectoral action, and HIAs. However, if health means everything it also means nothing, prompting the retort that if other sectors are doing HiAP work anyway it makes the additional emphasis on health redundant (2017: 888).

Some studies suggest ways to avoid health imperialism: contribute to a new ‘shared language’ across sectors ( Molnar *et al.*, 2016: 8–10); rebrand HiAP aims in terms of wellbeing, ‘living conditions’, ‘social sustainability’, ‘human rights’ or ‘civic participation’ to generate cross-sectoral ownership ( Synnevåg *et al.*, 2018b: 70–1; Scheele *et al.*, 2018: 64); and rebrand Health Impact Assessment (HIA) as ‘overall policy appraisal’ ( Kemm, 2001: 82–4; see also Adam *et al.*, 2014). However, as with win-win approaches, there is tension between seeking the benefits of collaboration and experiencing the ‘corruption’ of HiAP’s social determinants agenda ( Holt *et al.*, 2017).

*5. Identify policy champions and entrepreneurs*

HiAP studies describe the decisive impact of key individuals able to use their knowledge, networks, and skills to address multiple obstacles to HiAP progress. Some describe case studies of specific individuals, including:

- The Commissioner of the Minnesota Department of Health (US) was key to making sure that public health documents began with a discussion of social determinants. It helped reduce the time in meetings devoted to individual lifestyles. Meetings focused on ‘hard truths’ on issues such as ‘structural racism’ to avoid racial inequalities and marginalisation being subsumed under ‘inequality’ ( Bliss *et al.*, 2016: S91).
- Kickbusch *et al.* (2014: 187–92) describe Kickbusch’s impact as a ‘policy entrepreneur’ in South Australia. She helped convince policymakers that a focus on the social determinants of health could help reduce the unsustainable burden on health services (compare with the entrepreneurs in Iran who were effective in agenda setting but not wider collaboration - Khayatzadeh-Mahani *et al.*, 2016: 776–7; Khayatzadeh-Mahani *et al.*, 2019: 779).

Others focus on developing the skills to perform key roles. These roles can be almost synonymous with skills, including specialist ‘intermediaries’ and ‘relationship builders’ ( Kickbusch *et al.*, 2014: 187–92), or ‘stewards from the health sector’ who engage in other sectors ( Hendriks *et al.*, 2014: 175). In that context, Damari & Chimeh (2017: 407) describe many ‘specialized skills’ required of HiAP advocates, including:

‘ability to plan, public participation, intersectoral collaboration, social marketing, working with the media/media friendly attitude, advocacy, research management and knowledge translation, evaluation of health programs, network establishment and management, deployment and institutionalization, operational research, empowerment and consultation, and protocol and service pack design’.

Or, studies describe the importance of actors across political systems rather than senior elected leaders, including the ‘middle managers’ found in central and local government and public services ( Kranzler *et al.*, 2019: 2).

*6. Use HiAP to promote the routine use of HIAs*

The WHO defines HIA as ‘a combination of procedures, methods and tools by which a policy, program or project may be judged as to its potential effects on the health of a population, and the distribution of those effects within the population’ ( European Centre for Health Policy, 1999: 4; Finer *et al.*, 2005: 278). They inform political choices rather than providing a narrow technical process with a predictable output ( Finer *et al.*, 2005: 282; Kemm, 2001: 82–4). HIAs can help identify the value of non-health initiatives to reducing health inequalities ( Bert *et al.*, 2015: 45; Hall *et al.*, 2016: S50; Wernham & Teutsch, 2015). Their key elements are:

1. Analysing a draft policy proposal to assess its health and health equity effects
2. Structured dialogue across sectors and with stakeholders
3. Making recommendations
4. Flexibility of use, rather than used at a fixed point in time or stage of development ( Harris *et al.*, 2012: 4).

Harris *et al.* (2012: 5) describe the symbiotic relationship between HIA and HiAP:

- *HIA is a tool for HiAP*. HIA is a response to a new initiative (often after it has been proposed), where people cooperate to predict its impact on health/equity.
- *HiAP provides a ‘rationale for HIA’*. HiAP enables systematic engagement (often by a specialist unit) to identify opportunities for new initiatives and collaboration to gather evidence and generate recommendations for change ( Harris *et al.*, 2012: 5; see also Delany *et al.*, 2014: 3–4; Freiler *et al.*, 2013: 1069).

As such, HiAP commitment is associated strongly with HIA use. Their use *in principle* is established ‘in almost all highly developed countries’ ( Mattig *et al.*, 2017: 150), but *in practice* it ‘differs significantly between countries in regard to administrative and political structure, human and financial resources, and political will, support and commitment’ ( Mannheimer *et al.*, 2007: 307–8).

In that context, the WHO European Healthy Cities project helped raise awareness of social determinants *somewhat*, and encouraged *some* intersectoral working by boosting three elements of HIA:

- *Acceptability*. Demonstrate that: it provides economic benefit, there is stakeholder engagement, local stakeholders can use the tool flexibly, and it comes with a relevant evidence base and clear language.
- *Technical and political feasibility*. Foster politician and stakeholder support, early participation by partners, long term investment in the process, regular communication, and the sense that the systematic use of HIA brings something new to the process.
- *Sustainability*. Foster a long term vision, corporate level discussions, comprehensive tool development, the sense that health is embedded in each profession, and ownership among planners ( Simos *et al.*, 2015: 72; 80–1).

On the other hand, HIAs may be undermined by the absence of a HiAP strategy because they can be seen as a tool of ‘health imperialism’ that adds bureaucracy, or a luxury when funding is tight ( Gottlieb *et al.*, 2012: 158; Kemm, 2001; Oneka *et al.*, 2017). Examples of limited use include:

- The absence of legal mandates to use HIAs in Sweden prompted advocates to frame their use in terms of meeting another sector’s objectives and drawing on pre-existing partnerships ( Shankardass *et al.*, 2015: 469). Still, in local government, they were used mostly to facilitate healthcare contracts ( Finer *et al.*, 2005: 282).
- Although Canada tends to be a leader in public health policy, few Canadian federations used HIAs frequently ( Greaves & Bialystock, 2011: 406). One exception is Quebec, where HIAs are mandated and there is less need to make a win-win case ( Shankardass *et al.*, 2015: 469).
- The use of HIAs at the national level was opposed successfully by businesses in Switzerland, while *some* cantons (they studied 3 of 26) show that local government is more conducive to HiAP and HIA since they have a ‘pragmatic approach to partnerships and everyday policy orientations’ ( Mattig *et al.*, 2017: 154).
- Their use in Finland is ad hoc, and rarely to assess economic policies or to reduce health inequalities ( Melkas, 2013: 26)
- HIA as a tool for ‘evidence-based reasoning’ is ‘not very effective in the Netherlands’ ( Steenbakkers *et al.*, 2012: 289)
- HIA is a ‘box ticking’ exercise in the European Commission ( Franklin, 2016: 30).
- Simos *et al.* (2015: 72) generally found that policy actors in cities did not feel they had the resources for HIA.

*7. Do not rely on a traditional cost-benefit-analysis case for HiAP*

Advocates need to demonstrate HiAP’s economic value, such as to support economic policy aims in relation to lower overall costs, return on investment, or efficiency. However, it is difficult to make a short-term ‘business case’ for HiAP because:

‘(1) public health benefits are generally dispersed and delayed; (2) benefactors of public health are generally unknown and taken for granted; (3) the costs of many public health initiatives are concentrated and generate opposition from those who would pay them; and (4) public health often clashes with moral values or social norms’ ( Mayes & Oliver, 2012: 181).

Although economic arguments are important to win-win strategies, a HiAP business case requires different rules to justify investment ( Pinto *et al.*, 2015: 2–6). Most HiAP staff do not have the skills to conduct sophisticated cost-benefit analyses, and tend to rely on counterfactuals and “common-sense findings (‘reducing poverty must save money’) rather than on formal analyses”. As a result, the cost of implementing HiAP or the saving to government from upstream measures is unknown (2015: 6). A pragmatic option is to accept minimal additional funding for HiAP, and seek to incorporate it into existing budgets, but at the risk of preventive policies being treated as expendable (2015: 4–5).

## Updates and challenges to the HiAP playbook

Two emerging pieces of advice highlight the limits to the playbook and suggest that key elements should be amended.

***Treat HiAP as a continuous commitment to collaboration and equity, not a model***

The *Helsinki Statement* describes HiAP as a model with a ‘starter’s kit’ that ‘can be easily adapted for use in different country contexts and at the regional and global levels’ ( WHO, 2014: 7). This idea is not supported by HiAP research. Reflections on empirical studies suggest that HiAP is ‘abstract concept with rhetorical ideas’ which makes it challenging to convert ‘into practice and evaluation’ ( Huang *et al.*, 2019: 2). It ‘can be defined in different ways’ and ‘empirically it can mean different things’ ( Storm *et al.*, 2014: 184). As such, ‘every HiAP initiative is uniquely designed and governed, and so it is challenging to understand how to translate studies of one case to others’ ( Shankardass *et al.*, 2018a: 2).

Some studies have responded by trying to propose a new common understanding of HiAP ( Huang *et al.*, 2018: 25) or a ‘new model of intersectoral public health’ ( Bteich *et al.*, 2019: 242). However, these efforts symbolise an inherent flaw of such approaches, characterised by the initiation of a model that makes sense to a small group of specialists, followed by the discovery of ambiguity in collaboration with other actors, then an attempt to establish a new (but problematic) model. Each model is flawed because it focuses on what advocates need to happen to make HiAP models work, with limited adaptation to what actually happens in practice.

***Change the way you understand implementation***

This empirical work prompts us to revisit the meaning of HiAP implementation. For example, like most HiAP researchers, Freiler *et al.* (2013) initially define implementation as ‘The carrying out of a governmental decision as specified by official legislation or formal strategy (ie, mandate)’. Then, they emphasise the need for coproduction and intersectoral action. Each government cooperates with stakeholders to make sense of HiAP in a specific context, requiring it to coproduce the goals and actions that establish what would constitute implementation success. A government also adapts continuously to policymaking complexity and the tendency for policy to produce unintended consequences ( Shankardass *et al.*, 2018a: 3). The result is not described well by the phrase *HiAP implementation* if we use a narrow definition. Rather, studies now examine the high commitment to making sense of HiAP in context, which suggests that the outcomes are not specified in advance.

The best example is Corburn *et al.*’s (2014) study of Richmond, California. The Richmond experience of ‘coproducing health equity in all policies’ ( Corburn *et al.*, 2014: 624) provides lessons that few other HiAP initiatives can match, in which co-production methods help:

*1. Practitioners make sense of broad HiAP aims through the eyes of stakeholders/ citizens*.

They began with reference to WHO definitions, then focused on social justice in relation to income and wealth, which differs markedly according to race and immigration status (2014: 625)

*2. Produce priorities that were not anticipated in a desktop exercise or in interviews with practitioners.*

A key theme from discussions was the impact of structural racism on the daily activities crucial to health: ‘seemingly neutral policies and practices can function in racist ways by disempowering communities of color and perpetuating unequal historic conditions’ (2014: 628). Residents described social determinants in relation to environmental, social, and government problems: ‘in the same day, they might experience or fear violence, environmental pollution, being evicted from housing, not being able to pay health care bills, discrimination at work or in school, challenges accessing public services, and immigration and customs enforcement (ICE) intimidation’ (2014: 627).

*3. Non-health sector workers understand their role in reducing health inequalities*.

Staff in non-health sectors were described as ‘community clinicians’ to signal the impact of their work on health, while indicators of HiAP progress relate partly to the proportion of city employees who are women or people of colour (2014: 628–30).

***Take HiAP dilemmas seriously, focusing on the implications of collaboration and decentralisation for evaluation***

This recognition of the lack of a single implementable model is crucial to evaluations of HiAP progress, shifting from a general focus on the implementation gap towards reconsidering what policy success means in practice.

First, *dilemmas arise when key HiAP aims seem coherent in theory but contradictory in practice*. For example, Synnevåg *et al.*’s (2018b) study of policy in Norwegian municipalities identifies an inherent contradiction and dilemma, when Norwegian municipalities are venues to deliver national policy and formal local plans *and* represent community deliberation (combining quantitative data and service user experience):

- The rationality of *national to local implementation*, emphasising hierarchy and obligation, may undermine the rationality of *continuous local collaboration*, emphasising co-production, creativity, and tailoring policy to local circumstances.
- Much HiAP advocacy relates to getting public health language into formal strategy documents, often at the expense of dialogue with partners to foster ownership of that agenda.

Synnevåg *et al.* (2019: 2) relate municipal tensions to the HiAP ‘legitimising process’. Citing Suchman (1995), they describe HiAP legitimacy as the perception that it is ‘desirable, proper, or appropriate within some socially constructed system of norms, values, beliefs and definitions’. They identify four elements, noting that they may contradict or undermine each other:

1. *Regulatory legitimacy* describes the use of regulations to support HiAP as a policy. Translating HiAP into local mandates is a relatively strong feature (2019: 4).
2. *Cognitive legitimacy* describes the extent to which HiAP aims are well understood and taken for granted as appropriate. It is somewhat apparent in the term ‘living conditions’ used by one municipality, but generally people are unsure what public health is and if they are doing it as part of their work (2019: 6–7).
3. *Normative legitimacy* describes the relationship between HiAP and government norms, values and aims. It *seems* high because public health is high on local agendas, but rhetorical commitment accompanies low ‘ownership’, and few participants outside of health relate their vague HiAP commitment to their values, norms, or work (2019: 5).
4. *Pragmatic legitimacy* relates to a strategy to get the agreement of powerful interests to foster HiAP. Some see the benefit of a public health focus, but others address public health because they feel obliged (2019: 6).

In that context, Synnevåg *et al.* (2018a); Synnevåg *et al.* (2019: 2) warn against triggering professional identity-based reactions against public health interference. Further, a collective sense of HiAP legitimacy may require ‘inter-professional education programs’ or ‘socialisation processes, developing dual identities based on an understanding of interconnectivity and the complementarity of roles’ (2019: 8).

Second, *HiAP studies warn against top-down approaches*. Holt *et al.*’s (2018: 49) study of Danish municipalities warns against using government reorganisations to address implementation challenges. They describe a tendency to reform governance structures to address a lack of intersectoral action, but each measure has unintended consequences:

- The creation of central units undermined public health actors, since they were moved from collaboration with service deliverers (which suited their skills) to strategic work (‘foreign’ to them) (2018: 52).
- Intersectoral committees produced mixed experiences. Some were good for specific tasks, but interviewees describe resource-consuming meetings with little output. Rather than committees solving low inter-sectoral commitment, low commitment sucked the energy out of committees.

These reorganisations ‘tend to reproduce the organizational problems they are intended to overcome’, suggesting that ‘It is time to dismiss the idea that intersectoral action for health can be achieved by means of a structural fix’ (2018: 48). The few that worked related to other factors: committed people with problem solving skills, long term relationships, and supportive accountability measures. Reorganising structures to fit the HiAP model is less useful than trying ‘to manage the boundaries and structural silos which exist in any organization, e.g. by promoting awareness of their implications for public health action and by enhancing the boundary spanning skills of public health officers’ (2018: 56).

Overall, these empirical accounts represent movement towards a new understanding of political and policymaking reality. They take us some distance from the sense that HiAP models can be treated as technical exercises or puzzles to be solved with a playbook. To treat HiAP as a continuous commitment to collaboration is to reject treating it as a model to be adopted and implemented and accept that the outcomes should not be evaluated in traditional ways.

## Country studies: best case examples and cautionary tales

Most country studies report a major, unexpected, and disappointing gap between HiAP commitment and outcomes. These general findings are apparent in almost all relevant studies, including: *the US*, where it is difficult to find accounts of HiAP that go beyond vaguely described strategic commitments ( Bliss *et al.*, 2016; Gottlieb *et al.*, 2012; Hall *et al.*, 2016; Mayes & Oliver, 2012; Wernham & Teutsch, 2015); and *Canada*, in which studies describe a gap between its reputation as a leader in public health policy and the lack of progress in federations ( Greaves & Bialystock, 2011: 406; Kongats *et al.*, 2019; Nykiforuk *et al.*, 2019).

However, they stand out in the most-researched ‘best case’ examples where HiAP advocates would expect to find relative success in relation to:

1. High political commitment and strategic action (such as South Australia)
2. Political and economic conditions conducive to HiAP (such as in Nordic countries).

These studies find that HiAP strategies to challenge the status quo are overshadowed by (a) a far higher commitment to existing healthcare policies and the core business of government, and (b) state retrenchment. Further, the HiAP playbook has disappointing results, such as when the win-win focus leads to relatively powerless HiAP advocates giving ground but receiving little in return.

***South Australia: a best case centralised model***

South Australia (SA) provides one type of best case analysis. It is an exemplar of a HiAP model:

- *Established centrally*: incorporated formally at a strategic level, backed by public health legislation and/or a strategic HiAP plan, with high rhetorical political support.
- *Introduced in a supportive context*: ‘of social policy innovation’, ‘attention to social determinants and health public policy from 1980s’, and a ‘cadre of skilled staff’ with high knowledge of intersectoral action and senior-level experience of intersectoral action ( Baum *et al.*, 2019a: 6).

As such, it is unusually well researched and evaluated (accounting for one-quarter of our articles) and often treated as a model for others to follow, even though it is a single regional government (one of six states in a federal system) responsible for a population of 1.6m ( Fletcher, 2013). 

Evaluations suggest that high commitment to HiAP and intersectoral action improves processes but not health equity, particularly in a ‘neoliberal’ country during austerity and public service retrenchment. There is a major gap between the expectations associated with its programme logic and the recorded results. These results prompted reflection on how to conceptualise, design, and evaluate HiAP projects and reflect on its policymaking context.

HiAP design: identifying supportive factors

Table 3 suggests that HiAP success requires good working relationships between people across many sectors, working together continuously to define problems and identify solutions, backed by senior policymaker support ( Lawless *et al.*, 2018: 513–4).

**Table 3.** Factors supporting or undermining HiAP implementation in South Australia.

Supporting	Undermining
A well-resourced ‘centrally mandated unit’	Resource-constrained sectors focusing on their ‘core business’
Department of the Premier and Cabinet leadership, providing a clear ‘authorizing environment’	Lack of consistent senior manager support, such as the chief executives in other sectors held to account for other aims
‘Maintaining trust and credibility’	‘Collaborators failing to honour commitments [or] uphold agreed processes of group engagement’
‘Aligning HiAP with core business and strategic priorities’	Silos. HiAP represents an additional cost or requires a new technical language
Clear timelines and achievable milestones	Lengthy HiAP projects not in tune with (a) staff turnover or (b) the tendency of chief executives to change their minds

Source: adapted from Delany *et al.* (2016: 893–5)

Institutionalising HiAP at every stage of policy development: Health Lens Analysis

From 2008, HiAP was a key feature of the SA strategic plan, with ‘Health Lens Analysis (HLA)’ as its key mechanism:

‘The HLA is designed to shift the policy frame and inform policy at the conceptual stage rather than towards the end of decision-making processes, as is more typically the case with the traditional Health Impact Assessment’ ( Lawless *et al.*, 2012: S16).

There are five stages of HLA: ‘engagement, evidence gathering, generating, navigating and evaluating’ ( Lawless *et al.*, 2012: S16). Initial evaluations of three HLAs (water sustainability, regional migration, digital technology) suggest that they produced: ‘increased understanding by policy-makers of the impact of their work on health outcomes; changes in policy direction; development and dissemination of policy-relevant research; greater understanding and stronger partnerships between health and other government departments; and a positive disposition toward employing health lens analyses in future work’ (2012: S15).

Using HiAP to encourage policy changes in other sectors

The establishment of HiAP at a strategic level became conducive to the adoption of specific HiAP-friendly policies, particularly when newly elected (and unelected) policymakers were motivated to adopt them ( Kickbusch *et al.*, 2014: 187–92; see also Baum *et al.*, 2017). For example, Newman *et al.* (2016) describe the *Healthy Weight Project* as ‘a workable, evidence-based systems approach to increase commitment to practical and politically viable opportunities across government to address the non-health environments supporting healthy weight’ (2014: 44).

*Healthy Weight* became a HiAP priority from 2009 after it became clear that targets on obesity levels in the population would not be met despite evidence of intersectoral action (2014: 45). It had been difficult to identify evidence of effective policy interventions at the ‘upstream’ level (i.e. beyond social marketing and health promotion) (2014: 47). In that context, their ‘logic framework’ helped identify: influences on healthy eating and exercise, relevant policy areas (food drink production; infrastructure, community, education, employment, etc.), and opportunities for policy in relation to land use allocation, support for behavioural change, food reformulation, and a locally-grown food scheme. They used this framework to explore which departments could best connect it to their core business, such as the housing division’s ‘Environmentally Sustainable Design Strategy’ to encourage healthy food production and access to healthy food (2014: 49). They drew on 5 years of good intersectoral collaboration and face-to-face consultation with departmental representatives to clarify the benefits to them, while anticipating ‘the need to develop cross-cultural understanding and mutual respect’ and overcome a reputation of health actors for being “too ‘focused on health’ or being ‘health imperialist’”(2014: 48–55). They conclude (from the perspective of the health department) that ‘governments can develop a systems approach to obesity prevention’ and achieve ‘policy commitments’ (2014: 56).

On the other hand, van Eyk *et al.* (2019: 1159; 1170) describe state action constrained by limited responsibilities and the power of commercial food companies. Their work included a 1-year desktop analysis to develop recommendations followed by a focus on implementation. They then describe limited progress despite following the HiAP playbook: trying to avoid the perception of health imperialism, emphasising the co-benefits of action, and framing the problem and recommended projects in economic terms to maximise support (2019: 1169–70). This approach limited their options without prompting other sectors to reciprocate.

Van Eyk *et al.* (2020: 958) also identify the promise of child literacy programmes, particularly since there is relatively low need to persuade another sector of the benefits of collaboration. ‘Health and education sectors have long been seen as natural partners with mutually beneficial goals’, childhood literacy is a key driver in each sector, and staff in each sector have experience of working in (or with) the other. Collaboration is backed by high support for early years policy, a clear strategy to join health and education, entrepreneurs and intermediaries, relationship building, a HiAP unit, evidence gathering, a central mandate, and multiple pilots (2020: 962–4). As such, this experience can be interpreted in two different ways: as a best case of ongoing success from which to learn (2020: 969), or as a cautionary tale in which limited progress is puzzling under the circumstances, reminding participants that ‘without systemic approaches, the results are likely to be fragmented and unsustainable’ (2020: 970).

Cautionary tales from evaluation: support for process rather than outcomes

The 5-year evaluation (2012–16) of the SA HiAP programme identifies mixed fortunes. On the one hand, there is evidence of *high and sustained buy-in to the process of intersectoral collaboration*. Baum *et al.* (2019a) describe a ‘culture shift’ and ‘strong evidence of occurrence’ of:

- *Strategies*, to develop new ways to connect actors, encourage collective problem solving, and secure senior support for HiAP initiatives
- *Activities*, to foster policy ‘champions’ and ‘entrepreneurs’, relationships, teams, and a ‘central mandate for action’ backed by ‘accountability and reporting’
- *Impacts on processes*, to increase awareness of health equity and its social determinants, foster learning, widen policymaker perspectives, change policy agendas, strengthen alliances, boost HiAP capacity, and produce more awareness of each other’s language while reducing silos ( Baum *et al.*, 2019a: 6).

Participants across many government agencies ‘readily understood HiAP as providing tools for improving the process of intersectoral policy development’ ( Van Eyk *et al.*, 2017: 14–5). More people in more sectors appreciated the approach ( Baum *et al.*, 2019a). There is some evidence of intersectoral cooperation which helped to prevent ‘lifestyle drift in strategy’ (2019a: 1). The HiAP team built on political commitment from 2007, securing six (full-time-equivalent) staff with a mandate to work with other sectors, and with HiAP part of the strategic plan and intersectoral action as part of its targets (2019a: 2).

On the other hand, there was *limited support for equitable outcomes*. There is limited evidence of the use of intersectoral action to address the social determinants of health and improve health equity. Policy documents show a *reduced* emphasis on health equity from 2013 when ‘economic pressures resulted in the government narrowing its priorities to economic goals’ (2017: 1). Equity was not ‘core business’ in non-health sectors focused on service delivery. It was seen by interviewees as ‘a nice thing to do’ but dropped after state retrenchment or when some issues became off-limits ( Van Eyk *et al.*, 2017: 14–5; Delany *et al.*, 2014: 3–4).

HiAP seems like a rhetorical strategy that describes big ambitions without driving policy or shifting resources. The HiAP budget was 0.01% of the $5.8bn health budget ( Baum *et al.*, 2019a: 1), which symbolises its minimal impact on a desired shift of resources. Baum *et al.* (2019a: 6) describe ‘moderate’ impact on investment in social determinants and ‘improved performance against sectoral targets’, but find no evidence of impact on health equity. Overall, ‘SA HiAP can be judged to have made a modest contribution to actions likely to have improved population health in South Australia’ in relation to a state government with limited powers to address social determinants (2019a: 13).

Reinterpreting the playbook: HiAP as the alternative to radical change?

The SA experience suggests that HiAP supported the agenda of policymakers who were not interested in health equity, or more interested in economic frames. Economic redistribution was not part of the government agenda, and the ‘broader underpinning factors dictating the distribution of power, money and resources were not addressed by HiAP’ (2019a: 1). In that context, of an imbalance of power towards non-HiAP actors, *intersectoral action may undermine HiAP*. A focus on avoiding health imperialism, seeking win-win strategies, and supporting government priorities helped maintain the status quo (2019a: 1). Further, the ambiguity of health equity is exacerbated when so many sectors with their own ideas are involved. SA’s HiAP team found that the word equity ‘”did not resonate” with other agencies’: some saw it as health jargon, and it did not translate into things like funding indicators; some equated equity with equal access to services; and, HiAP work took place in a ‘neoliberal’ government more likely to focus on individual action (2017: 3; 13; 16–20).

In other words, evaluations describe culture change and success in relation to intermediate steps, rather than how to distribute resources to address policy problems in new ways. At times, these intermediate changes seem to represent an *alternative to more radical change*, to support the agendas of others, producing minimal challenge to the ‘neoliberal’ economic agenda accentuated by austerity measures. If so, there appears to be a large and growing gap between the programme logic as described at the beginning of the process and the outcomes described by the end. In the beginning, the idea was that the best way to tackle social determinants would be with new collaborative policy processes as if collaboration would foster cultural change (akin to the argument that people will change their minds or do things differently if they have better information provided by skilled champions). By the end, it appears that such a strong focus on pragmatism (foster consensus, avoid health-imperialism, seek win-win outcomes) provides a government with cover: they can use the language of radical change in *policy processes* as an alternative to radical changes in *policy instruments*.

## Nordic Countries: best case decentralised models

Nordic countries represent exemplars of decentralised or ‘community’ HiAP models, in which central governments provide the authorising environment (strategy and legislation), funding, and research support, but local governments are responsible for their own HiAP strategy and implementation ( Carey *et al.*, 2014: 3). Three countries - Finland (10), Norway (10), and Denmark (7) - account for one-quarter of HiAP articles. They represent a different type of best case scenario, in which they have shown HiAP leadership and provide a relatively conducive context in relation to high levels of welfare state provision. As such, their experiences highlight the irony of rising rhetorical commitment to HiAP undermined by political and economic changes that made each country’s context seem less conducive to HiAP aims. 

***Finland: a test case for decentralised HiAP***

We would expect Finland to provide positive lessons for three reasons. First, it was a ‘pilot country for the World Health Organization’s Health For All (HFA) strategy in 1986’ ( Kokkinen *et al.* (2019a: 259). Second, it is one of ‘the Nordic Social Democratic welfare states’ that were initially the most willing to curb international capital, foster equity, and address poverty (2019a: 260). Third, it has a long history of decentralised public health innovation and a ‘culture of collaboration and societal values’ ( Puska & Ståhl, 2010: 315–20; Ståhl, 2018: 39). Approximately 310 municipalities (total population 5.5 million) are ‘responsible for social services and health care, basic education, upper secondary education, town planning, the technical infrastructure, environmental protection, culture and sport’ and they work with central government to foster policy learning ( Melkas, 2013: 4). For example, the North Karelia project helped reduce population cholesterol levels via community reforms encompassing a shift towards greater food and vegetable consumption and less dairy fat, and was scaled up nationally ( Puska & Ståhl, 2010: 315–20). In other words, Finland was an early HiAP adopter, shifting from a focus on individual lifestyles towards an ‘ecological approach’ to social determinants, and key to raising HiAP on the EU agenda during its Presidency in 2006 (2010: 322–5; Ollila, 2011: 12–3).

Finland as a cautionary tale: the role of political economy

Finland’s conduciveness to HiAP diminished over time. From the 1990s, it often elected governments more open to the market and less willing or able to maintain Finland’s low Gini coefficient or labour protection laws, especially after it entered the EU (2019a: 260). Further, the Ministry of Finance took on a greater role that could undermine HiAP, by limiting budgets to municipalities and encouraging them to rationalise services (such as school nurses and home helps) and use the private sector more ( Kokkinen *et al.*, 2019a: 261). Municipalities from 1993 were freer to act, but with less money, prompting high variation and poor outcomes in response. Kokkinen *et al.* (2019a: 258; 263–4) note the impact of the ‘changing role of the state’ and ‘welfare state restructuring’ on the implementation of public health programmes, including reduced funding allocations plus market deregulation and pro-growth policies. Similarly, Shankardass *et al.* (2018a: 6) find that economic and health sectors had different objectives, and the government had a longstanding commitment to ‘neoliberal’ ideas (i.e. focused on individual responsibility), which undermined the HiAP focus on social determinants.

HiAP as an implementation problem

Finland’s experience highlights a continuous delivery problem. At a national level, initial evaluations highlighted limited implementation, partly because the HiAP strategy was too centralised and health-driven:

‘The programme had been drawn up largely by health experts and written in the language of planning, and the public was not well-informed of the program as a whole. The main bodies responsible for the decision-making had not adopted a permanent role in the implementation of the programme, and there was no monitoring mechanism set up to provide an assessment of the influence of other sectors on health’ ( Melkas, 2013: 6)

This experience prompted a revised approach that ‘relied a lot more heavily on cooperation between the state, the local authorities and organizations’ (2013: 6). Still, the use of key measures – such as HIAs - remained ad hoc, not used enough to assess economic policies, or to reduce health inequalities substantially, while smoking, alcohol consumption, and unhealthy eating remained major problems (2013: 26). This limited progress is apparent despite evidence of long-term intersectoral action.

HiAP implementation at local levels: the measures are not promising

The ‘Finnish Benchmarking System for Health Promotion Capacity Building (BSHPCB)’ is the main tool to assess local implementation ( Ståhl, 2018: 43). It was launched in 2010 after 4 years of work, to provide seven ‘comparable, objective indicators for the management, planning, and evaluation of health-promotion activities in municipalities’. So far, they show minimal evidence of projects having a substantive impact on health equity.

***Norway: a leading country providing (often negative) lessons***

Studies of Norway as a HiAP leader highlight the importance of political and economic context. Fosse & Helgeson (2017: 1) describe a largely supportive context in which the Norwegian social democratic state reflects its historic commitment to address the social determinants of health (although some governments oversaw more individualistic health promotion). As such, it fosters key elements of HiAP, including: high commitment and attention to ‘health promotion’, a focus on the ‘broader determinants of health’ and ‘to reduce social inequalities in health’, a recognition of the need for multi-level and multi-sectoral collaboration, and ‘action plans with concrete targets, deadlines, and responsibilities’ ( Fosse, 2011: 266–7). 

There are three key elements. First, a *relatively strong commitment* to address health inequalities via income redistribution ( Fosse, 2011: 267). Second, the *national role is strategic, regulatory, and exhortative*. The Public Health Act (PHA) in 2012 ‘places the responsibility for public health work as a whole-of-government responsibility rather than a responsibility for the health sector alone. Municipalities, county authorities, called county municipalities (CMs), and central government authorities are all considered important actors in the efforts to promote public health and reduce social inequalities in health’ ( Fosse & Helgeson, 2017: 2). The PHA established a central coordinating role for a decentralised system ( Hagen *et al.*, 2018: 808).

Third, *HiAP-driven local governments*. There are 428 municipalities of varying size, delivering many of the services (including welfare) associated with HiAP. The PHA obliges them to incorporate public health in planning and administration ( Fosse & Helgeson, 2017: 2). Earlier central government initiatives suggested that ‘municipalities establish the position of public health coordinator (PHC)’ ( Hagen *et al.*, 2015: 598). Municipalities are ‘are agents for the welfare state, implementing national policy goals’ and ‘they form local independent democratic arenas to meet local preferences and needs’ ( Synnevåg *et al.*, 2018a: 68). Local leaders are expected to choose how to foster public health (2018: 69), supported by chief administrative officers ( Hofstad, 2016: 569). They are encouraged to research the health profile of municipal populations (to produce a ‘health overview’), identify and target ‘underserved groups’, consider all policies in light of their ‘public health impact’, and appoint a PHC ( Hagen *et al.*, 2018: 807–8).

Norway as a cautionary tale: limited local development, resources, and agreement

Hagen *et al.* (2018: 808) identify several difficulties. Municipal actors are unsure about key elements, such as: how to produce health overviews in relation to complex problems, or to cooperate across multiple departments (especially when only one is enthusiastic), and engage with debates on the extent to which their response to equity should be universalism or ‘proportionate universalism’, or to pursue social determinants or individual lifestyle-driven approaches (2018: 813–14).

Limited local development.

Initial surveys of 19 county municipalities (2011–12 and 2014) found that few adopted HiAP strategies. Most focused on ‘diet, physical activity and other lifestyle issues’ ( Fosse & Helgeson, 2017: 3; 8) Five expressed a reduction in health inequalities as an aim. The PHA gave county municipalities a way to encourage others to work with them, but is not a substitute for national direction on how to collaborate. Silos remain, and joined up government is elusive (2017: 8–9). The same research team’s larger survey suggests that most municipalities (70%) considered the issue of fair resource distribution when pursuing health strategies, but fewer (38%) extend this approach to local policymaking in general ( Hagen *et al.*, 2018: 811). While the ‘process of developing a health overview seems to build the institutional muscle, awareness, and skills among relevant municipal personnel to address health inequalities’, 58% do not engage in it (2018: 813). As such, key goals are ‘too vaguely defined and uncoordinated’ in municipal plans ( Hofstad, 2016: 571–4)

Limited municipality resources for research.

Hofstad’s (2016: 570) respondents describe their limited resources to produce public health overviews (the evidence base for practice), or find good evidence on health inequalities. Many local actors prefer to focus on ‘vulnerable groups’ rather than ‘health equity’, and there is a mismatch between general HiAP aims and specific planning activities and ‘statutory provisions’. PHCs are generally part-time and too junior to foster HiAP effectively ( Hagen *et al.*, 2015: 601; Hagen *et al.*, 2018: 807).

Limited agreement on key terms.

Synnevåg *et al.* (2018a: 68) argue that public health terminology may hinder local implementation. Their interviews suggest that: (1) ‘public health work’ is defined so broadly as to mean everything and therefore nothing; (2) there seems little benefit to describing policies for wellbeing as ‘public health’, (3) renaming activities as ‘public health’ is an exercise in health imperialism; and (4) the branding may be good for legitimacy initially, but should be dropped later (2018: 70–1).

***Denmark and Sweden: high support, low impetus for local action***

Multiple studies of Denmark (and some of Sweden) suggest that Finland and Norway’s limited progress reflects a Nordic experience. Scheele *et al.* (2018) identify the gap between progressive Scandinavian (Denmark, Norway and Sweden) welfare policies and actual results on health and wealth, including the ‘lack of success of public health efforts to improve health of citizens with low income and limited education’ (2018: 57). They contrast the high importance of local government to HiAP versus the low importance of HiAP in municipal government (2018: 58). Their interviews identify:

1. High but vague commitment. There is low interest, insufficient use of political commitment to cross sectoral divides, and no good way to demonstrate that HiAP is a good use of the annual budget.
2. A tendency for the health unit to have responsibility, with low incentive for other sectors to take the initiative or support wider aims such as ‘social sustainability’.
3. Multi-level tensions when municipalities rely on national/ regional governments for data and guidance but seek autonomy and locally tailored approaches.
4. The importance of knowledge of the problem to boost political support, undermined by limited evidence on solutions.
5. The lack of a ‘common language’ to monitor implementation, albeit addressed by Sweden’s national government when providing indicators of ‘health *determinants* rather than health *outcomes’* (2018: 59–64).

They conclude that there is no ‘showcase municipality’ showing good results. Such conclusions are backed by single-country studies in Denmark. Christensen *et al.* (2019: 216–8) explore the prospects of ‘Health in All *local* Policies’ in Husum, Copenhagen (Denmark), finding a gap between common expectations and actual experiences. The organisers and participants were committed to a local collaborative network and were aware of key facilitators (2018: 218), but had little stake in it, fostering more ‘communication and coordination than collaboration’ (2018: 227).

Further, Holt *et al.*’s (2018: 49; 2017: 881–2) study of Danish municipalities identifies ‘the tension observed between a general popularity of intersectoral policymaking for health and the great challenge posed by its implementation’. Municipalities have responsibility for local public health and a range of services relevant to HiAP (2018: 49). The Danish health act emphasises the opportunity for municipalities to think holistically about public health and encourage intersectoral action. Yet, few municipalities take this opportunity. Sectors such as education focused on the causes of ill-health (e.g. unhealthy eating, tobacco, alcohol, low exercise) and the idea of ‘healthy schools’ rather than the causes of the causes (e.g. educational progress) (2017: 888). Health managers, committed to emphasising that health would be a vehicle to achieve another sector’s objectives or cut costs, struggled to frame such work in relation to upstream measures (2017: 888; see also Andersen & Gulis, 2017; Larsen *et al.*, 2014)]

## From Nordic to European experiences: limits to the HiAP playbook and national and local ‘maturity’

Nordic experiences provide some of the most challenging conclusions to the HiAP playbook. Their status as HiAP leaders, in countries conducive to the HiAP agenda, ensures that these lessons provide internationally relevant cautionary tales. Further, their combined experience qualifies the often-expressed expectation that local government is more conducive to consensus-driven intersectoral HiAP work ( Mannheimer *et al.*, 2007 on Sweden; Mattig *et al.*, 2017 on Switzerland; De Leeuw & Clavier, 2011 on the Netherlands)

This lack of progress is also a feature of EU studies: ‘Since the 2006 Finnish EU presidency, HiAP is regularly referred to by the Commission, but has not yet been implemented as an overarching political vision’ ( Godziewski, 2020: 1307). Further, while there are fewer studies of other European countries, those that exist tell a similar story (which we explore in more depth in Cairney *et al.*, 2022). Most notably, Storm *et al.* (2014); Storm *et al.* (2016) develop a measure of HiAP ‘maturity’ ( Table 4) and apply it to the Netherlands.

**Table 4.** Storm *et al.*’s stages of HiAP maturity.

Stage of maturity	Description
0 - ‘Unrecognized’	‘there is no specific attention for the problem, in this case the problem of health inequalities’
1 - ‘Recognized’	‘municipalities recognize the problem and the solution of HiAP and there is clarity which activities will alleviate the problem’
2 - ‘Considered’	‘there are preparatory HiAP actions on parts of the problem. For example, HiAP is described in the local health policy document as a means to reduce health inequalities, collaboration between health and non-health sectors is started (project-based), and there are preparatory actions and activities to influence determinants of health inequalities’
3 - ‘Implemented’	‘HiAP investments in several problem areas exist. Non-health sectors are involved in the policy making process as well as in the process of policy implementation to reduce health inequalities. Collaboration agreements are made between sectors. Structural consultation with others sectors and the presence of a key person for HiAP are available’
4 - ‘Integrated’	‘quality processes are an integrated part of HiAP. There is a broad, shared vision on how to reduce health inequalities by HiAP, and there are visible milestones (both content and process)’
5 - ‘Institutionalized’	‘there is a systematic improvement of HiAP quality. There is political and administrative anchoring of the HiAP approach and HiAP is considered at every municipal policy cycle’

Source: Storm *et al.* (2014: 183)

Their application of this measure to municipal government highlights HiAP *immaturity*. Of their sample of 50 municipalities, only 16 are active enough to participate, including three at stage 1, seven at stage 2, four at stage 3, and two at stage 4 (2014: 186). Peters *et al.* (2016: 291–4) report similar results from a national public health programme encouraging HiAP approaches (2009–15), with a tendency for municipal governments to pursue health communication and individual lifestyles. Steenbakkers *et al.* (2012: 293–4) report the failure of their coaching programme to convince policy managers of the payoffs to long term intersectoral action. Overall, the Dutch experience reinforces HiAP’s limitations in practice, in which there are only proxy measures of success, limited knowledge of what is going on in practice, and with no sense that HiAP ‘maturity’ would cause greater health equity.

## Multiple country studies

Some studies synthesise lessons from multiple countries ( Jackson *et al.*, 2006; Molnar *et al.*, 2016; Pinto *et al.*, 2015; Shankardass *et al.*, 2015). Two consistent lessons emerge. First, HiAP adoption relates strongly to context, including ‘cultural, economic and political’ factors, ‘international influences’, the size of the health inequalities problem, levels of previous intersectoral experience, and the ideology underpinning the adoption of HiAP ( Shankardass *et al.*, 2015: 466). Second, there is a large implementation gap regardless of the political system.

For example, Friel *et al.* (2015) examine HiAP developments in the Western Pacific Region: 37 countries with a total of 1.8 billion people. Their review highlights the importance of a common narrative of HiAP, political will and senior leadership to foster intersectoral action, and the role of previous efforts and health promotion capacity (2015: 326–8). They describe a ‘building blocks’ approach emphasising familiar HiAP playbook elements:

- Framing public health issues in relation to different audiences.
- The need to shift from a ‘curative’ to a ‘social determinants’ agenda emphasising whole-of-government approaches (‘this is everybody’s business’).
- The need to avoid calling HiAP new, focusing instead on the strengths of each country (e.g. ‘among Pacific Island countries, there is a long history of a broader inter-disciplinary approach to tackling health issues’, 2015: 330)
- Building on existing relationships between health and non-health ministries and stakeholders, NGOs, community groups.
- The importance of the private sector to some initiatives (e.g. on helmet laws).
- A combination of formalised support and informal collaboration.
- The need for financial support (2015: 328–31).

They describe the importance of health equity as a driver, but are unsure how many HiAP strategies come with equity targets (2015: 331–2). As usual, health equity ‘needs more explicit attention’, and clarity on whose equity counts (2015: 333–4).

Overall, these regional and country-level experiences identify a major gap between (a) initially high expectations for policy change and policymaking reforms, and (b) actual policy and practices.

## Comparative studies of local or municipal government

Two reviews identify comparative insights from the study of local government. Guglielmin *et al.* (2018) highlight contextual variations (mostly in Europe and Australia) that explain conduciveness to HiAP progress, including: *the type of political and economic system*, the extent to which health equity was already a focus (equity is not a big feature of the reviewed articles), and local geographical factors (including population size). They list commonly described factors related to implementation, largely suggesting that the *absence of these factors helps explain limited implementation* (2018: 287–90):

1. adequate dedicated funding
2. a ‘shared vision across sectors’
3. ‘national leadership’ backed by legislation
4. a mechanism for local ‘ownership and accountability’ to set clear expectations
5. ‘local leadership and dedicated staff’
6. an effective HIA process to facilitate coordinated action with non-health sectors
7. ‘local health and policy process indicators’ (to compete with healthcare targets).

Van Vliet-Brown *et al.*’s (2018) scoping review identifies a common HiAP story but also key differences between North American and European HiAP governance. Studies have 5 main foci when discussing HiAP adoption (2018: 716):

1. Governance differences suggest that *HiAP champions* are more likely to be found in government in Europe but community-based organizations in the US (2019: 719).
2. *Trigger issues* include health crises (an NCD epidemic) and non-health issues including ‘violent crime, transportation and safety, pollution, cumulative environmental stressors and extreme weather events’ (2018: 717)
3. The importance of *intersectoral collaboration*.
4. *Facilitators* include ‘stable funding mechanisms’, ‘strong long-term political support’, ‘open vertical and horizontal communication channels’, ‘effective public engagement mechanisms’, ‘established taskforces’, ‘and legal obligations’ (2018: 718).
5. *Barriers* include ‘lack of stable funding’, ‘lack of a clear vision or objectives’, ‘intersectoral collaboration perceived as an extra task’, ‘inadequate understanding or expertise’, ‘siloed organizational structure’ (2018: 718).

Recommendations for HiAP implementation include: ‘supportive government structures, collaboration and personal actions’, including more training for municipal workers, establishing a ‘HiAP taskforce’, including HiAP in high level strategy documents, good communication, budgets, ‘engaging all departments early on in policy development’, collaboration with actors outside government (including lawyers in the US) (2018: 718).

However, the reviewed publications do not demonstrate the benefit of HiAP in these settings and do not provide good evidence on the impact of alleged facilitators (2018: 719–20). Rather, they identify a tendency for policymakers to reach for a HiAP approach reactively, such as following a social or environmental crisis (2018: 20). Or, they focus on governments or units that are taking the HiAP lead, adopting a program logic, and telling the usual story about ‘evidence-informed decision-making to meet the challenges of creating healthier and more productive communities’ (2018: 719–21). This conclusion does not resolve the key issues identified by Van Vliet-Brown *et al.* (2018: 719–20):

1. *Ambiguity*. They contrast a (a) relatively clear definitions and lists of implementation actions in the *Helsinki Statement* with (b) low clarity on the meaning of HiAP in the literature or in practice. HiAP may be described either as a fully formed model, ‘formalized process’, ‘shift in the underlying philosophical paradigm of society’, ‘abstract concept’ that is difficult to implement, or synonymous with the use of HIAs.
2. *Structure versus flexibility*. The need for a balance in which HiAP ‘is structured in a way that municipal decision-makers can easily see the value of integrating it into their organizational culture while keeping it broad enough to allow for contextually informed implementation strategies to be developed’ ( Van Vliet-Brown *et al.*, 2018: 719–20)

## HiAP studies beyond the Global North

This combination of foci – on the HiAP playbook and country studies – helps identify a *saturation point* in which no study provides new information to supplement or challenge our findings. However, we reach this point partly because a small number of countries inform empirical assessments of HiAP models and they are generally in the Global North. Most studies of countries in the Global South provide similar messages (with the exception of Cuba), but in a context with great potential to change the meaning of their findings.

***Iran.*** Early experiences in the Kerman province highlight the importance of a HiAP mandate and policy champions, but low ‘maturity’ of the HiAP network. There is a major gap between (a) centralised systems and the hierarchical nature of existing networks compared to (b) HiAP ambitions for decentralised and collaborative dynamics ( Khayatzadeh-Mahani *et al.*, 2019). Khayatzadeh-Mahani *et al.* (2016: 776–7) describe no national health improvement (over 10 years) and a HiAP plan ‘doomed to failure’, based on ‘poor formulation’ (including limited consultation by key entrepreneurs, low collaboration, low resources) and low political commitment (2015).

***Kenya.*** Mauti *et al.* (2019: 1) identify the gulf between (a) high-level commitment to HiAP, but (b) minimal follow-through: ‘the implementation of intersectoral action focusing on health promotion is still arbitrary’ and HiAP ‘is still perceived by many stakeholders as the business of the health sector, rather than a policy for the whole government and beyond’. The social determinants approach is not well known, there is little sense of how to implement HiAP, evidence of cooperation across departments is minimal, and high level support depended strongly on a former Minister of Health (2005), Hon. Charity Ngilu (2019: 5–9). Adopting HiAP in principle has produced minimal action in practice.

***Cuba.*** Baggott & Lambie (2018) suggest that Cuba performs disproportionately well. Its population is healthier than countries with its economic profile, and Cuba performs many functions sympathetic to HiAP: there is universal access to healthcare, focused on primary care, with doctor-nurse teams who perform regular checks, to ‘emphasize prevention, health promotion, and a holistic approach to health and wellbeing' and 'document individual risk factors, such as family history and lifestyle, and wider socioeconomic and environmental determinants, such as housing' (2018: 214). Yet, most countries treat Cuba a a 'celebrated anomaly' with few transferable lessons (2018: 213).

While most of these experiences are familiar to HiAP advocates, Byskov *et al.* (2019) situate them in historical political economy phases that are discussed rarely in Global North articles. By discussing Global South experiences, they highlight the wider paradigmatic and global political factors that provide a context in which to consider any public health initiatives, including:

1. A colonial era up to 1960, prioritising hospitals and hygiene.
2. A 1960s GN focus on improving GS national health systems.
3. A 1970s ‘new governance’ and systems thinking focus by the WHO.
4. A late 1970s global commitment to primary healthcare.
5. 1980s ‘donor dominance’, in which international organisations undermined national autonomy to pursue public health.
6. 1990s GS governments undermined by the conditionality of economic support by international organisations, focusing on healthcare efficiency and performance.
7. From the 2000s, the Millenium Development Goals are important, but in a ‘neoliberal’ context in which many organisations competed to provide funding and direction (and focused primarily on infectious disease).
8. Late 2000s systems thinking and the search for universal healthcare.
9. The 2015-onwards focus on Sustainable Development Goals

As such, their distinctive recommendation is to promote HiAP approaches built on local autonomy - ‘sub-national democratic priority setting for health action by a major scale up of participatory approaches supported by mutually accountable decision-making process guidance’ – to reduce GN interference (2019: 643).

## The use of policy theories in HiAP studies

Some HiAP studies use policymaking research to encourage and evaluate HiAP success. They identify the limits to HiAP, contrasting (1) the strong evidence on social determinants and a long tradition of health promotion thinking, with (2) slow movement towards addressing this problem and limited evidence of intersectoral action ( Baum *et al.*, 2019b: 834). Most studies focus on Australia, emphasising the political economy context where the dominant ideology is ‘neoliberal’ and individualist ( Baum *et al.*, 2019b: 839). Examples of ‘unfamiliarity, discomfort and racism’ exacerbate these problems’: some oppose HiAP initiatives on the spurious grounds of special treatment for indigenous populations affected disproportionately by social determinants approaches (2019b: 838). In that context, there are two main uses of policy theory ( Cairney *et al.*, 2021).

***Policy theories for practical lessons***

Three key studies provide agency-centred lessons for instrumental purposes (see also Ameratunga *et al.*, 2016). They perform a balancing act, to:

1. Extract lessons from theory-informed case studies of policy change, while acknowledging slow policy change.
2. Provide agency-centred lessons to improve the HiAP playbook, while acknowledging the limits to agency in policymaking systems.

Townsend *et al.* (2020: 2) draw lessons from national paid parental leave (PPL) which reduces health inequities via child health and ‘improvements in the health of women who are low paid or on contingent employment contracts’. Australia was the last OECD country to adopt it (2009), because it lacked a Nordic-style welfare state or commitment to gender equality (2019: 8–9). This context helps explain the relationship between framing and influence. Historic policy focused on the male breadwinner, defining parental leave in relation to industrial relations. Employers and industry associations opposed policy since they would bear the costs (2019: 3–4). HiAP-informed policy change was fostered by:

1. Growing alliances (beyond trades unions and feminists), framing parental leave in relation to economic, health, and gender equality benefits.
2. Pursuing a government rather than employer scheme (which diluted industry opposition and gained support).
3. Lobbying ‘different policy venues’ and
4. ‘the election of a socially progressive government as a key window of opportunity’ (2019: 5).

Framing strategies helped counter the actors who (a) argued that women should not be in the workforce if their children are under 5 (hence a focus on child benefits - 2019: 6), or (b) prioritised funding for low paid workers. PPL advocates emphasised health evidence, but were also pragmatic, to:

- Reduce demands, from 26 to 18 weeks paid leave.
- Exploit rather than challenge existing frames. They (a) related PPL to economic productivity to present a ‘business case’ commanding industrial support, and (b) accepted the rhetorical value of tying PPL to the health of mothers and children rather than parental equality (2019: 7–8).

This strategy was ( *eventually*) a ‘game-changer’, prompting Townsend *et al.* (2020: 9) to extract advice:

‘Our analysis highlights the benefit of deploying multiple synergistic framings, building coalitions with non-traditional policy allies and using multiple policy venues. This is likely especially important when the dominant policy concern is economic and when public health actions directly confront private sector interest groups’.

Harris *et al.* (2018: 1090) explain why health promotion gained a foothold ‘in legislation arising from land-use planning system reform in New South Wales, Australia’. Their analysis draws highly on policy theories to describe policy entrepreneurs exploiting an opportunity caused by the sudden perception in government that (a) the economic framing of the reform had fewer supporters and more opponents than expected, and (b) a focus on health benefits (reduced traffic jams and pollution) boosted support while being unthreatening to most actors. They use this experience to improve the HiAP playbook:

- ‘Be ready to recognize and exploit windows of opportunity’
- ‘Build a broad coalition of interested actors’
- ‘Know the main entrepreneurs and coalitions’
- ‘Where possible, be non-threatening and co-opt their support’
- ‘Ensure your issue and goal are prominent in the policy process’ (or ‘If it is not prominent, try to slip it in under the radar’), and
- ‘If necessary, challenge the policy monopoly’ (2018: 1098).

Khayatzadeh-Mahani *et al.* (2018: 4) focus on obesity policy, using a wide range of policy theories to enhance HiAP playbook strategies:

1. reframe obesity as a systemic not individual problem,
2. align this framing with the policy goals of other sectors (‘co-framing’), and
3. create a ‘network administrative organization’ (‘a separate legal administrative entity’) to facilitate collaboration and co-framing (2018: 8).

***Policy theories to improve programme theory and evaluation***

Some studies use policy studies to inform evaluations of HiAP progress. Storm *et al.* (2014); Storm *et al.* (2016) draw sparingly on policy theory, *assuming* that HiAP would work as intended if implemented, focusing on the measures to detect HiAP maturity. For example, ‘Health inequalities are reduced if’:

1. ‘several policy sectors collaborate in addressing such inequalities and/or their determinants’
2. ‘such inequalities and/or their determinants have priority in policy sectors and their reduction contributes to a shared objective’
3. ‘there is coordinated use of policies and activities across various policy sectors’
4. ‘multiple policy sectors regard the same issues as important and there are formal collaboration strategies relating to those issues’
5. ‘there is more intensive collaboration amongst policy sectors and if positive factors are favourably influenced’ (2016: 11; see also Freiler *et al.*, 2013: 1069).

They also produce *expectations* about the impact of specific measures. Storm *et al.* (2011) use interviews with officials to identify the ‘resolutions’ with ‘potential impact on health inequalities (and their determinants)’. They include better financial support, employment opportunities, physical, living, working, and social conditions, lifestyles, and education support for ‘vulnerable groups’ or their children, plus improvements in public health and public services.

The SA HiAP evaluation goes further, using policy theories to inform the programme theory underpinning HiAP design, delivery, and evaluation ( Baum *et al.*, 2014a: i135). They identify ‘how and why a program or policy is thought to work’, including three strategies that foster effective working:

1. ‘developing relational systems that connect individuals, agencies and sectors’
2. ‘undertaking joint problem identification and problem-solving’
3. ‘utilising governance systems that connect HiAP work with senior decision-makers’ ( Lawless *et al.*, 2018: 511–14).

Six commentary articles on Lawless *et al.* (2018) raise four main problems with this approach. First, there remains uncertainty on what HiAP actually is – a tool, objective, intervention, or mode of governance – and therefore what the programme theory seeks to explain ( Holt & Ahlmark, 2018: 759; Peña, 2018: 761). Second, there is confusion about how policy theories contribute to programme theories. Lawless *et al.* (2018) help visualise complexity without showing clearly how each action causes outcomes ( De Leeuw, 2018: 763–4; Harris, 2018; Holt & Ahlmark, 2018: 758; Shankardass *et al.*, 2018b). Third, there is a gulf between the assumptions underpinning HiAP theories of change and empirical studies. The former suggests that the pursuit of intersectoral action, built on win-win strategies and avoiding health imperialism, will foster more collaborative policymaking, better policy, and health equity. The evidence does not support these assumptions. Rather, it provides proxy measures of progress, including *commitment* to intersectoral action (see also Tang *et al.*, 2014; Tervonen-Gonçalves & Lehto, 2004; Wepner & Giesecke, 2018). 

Fourth, although HiAP is dedicated to challenging inequalities of power, programme theories remain technical exercises. Research focuses on safe issues like collaboration rather than ‘politics, power and ideology’ ( Oneka *et al.*, 2017; Raphael, 2015: 381). Few analyse political economy to highlight the power imbalances and dominant ideologies that undermine HiAP ( De Leeuw, 2018: 765; Harris, 2018: 875; Holt & Ahlmark, 2018: 758; Labonté, 2018: 656; Peña, 2018: 761; Shankardass *et al.*, 2018b; 757; although see Kokkinen *et al.*, 2019a: 258–9). De Leeuw & Clavier (2011: 237–40) and ( Clavier, 2016) argue that this low understanding of politics *undermines policy progress* by expecting intersectoral action to produce collaborative problem-solving rather than competition. Yet, policymaking involves a spectrum from collaboration to coercion ( O’Flynn, 2016: 440).

In that context, De Leeuw & Peters (2015; see also Hendriks *et al.*, 2016) identify nine questions to foster a HiAP playbook informed by political science:

1. *How has the problem been framed and by whom?* While scientists often focus on producing uncertainty, politics is about the exercise of power to reduce ambiguity (to ensure a preferred interpretation of policy problems).
2. *Which policies are already in force or in development?* New HiAP initiatives cannot be introduced in a vacuum. They interact with existing policies.
3. *What information is there about the problem, its magnitude and consequences, and relevant stakeholder positions?* Politics includes whose evidence counts when defining problems and choosing solutions.
4. *What facts, ideas and assumptions constitute the policy logic in relation to the problem?* Ask if HiAP initiatives are consistent with the values and assumptions of policymakers.
5. *What evidence, experience and opportunities exist to develop winning alternative approaches?* Note the competition to propose technically and politically solutions.
6. *What social, economic and institutional ‘win–wins’ can be established?* Each organisation may collaborate if the benefit (to its own goals) is clear.
7. *What are the power, priority and support positions of all stakeholders in particular policy proposal?* Produce a map of powerful actors to identify the potential for alliances.
8. *What politics are involved in the initiation and final stages of policy development and adoption?* Note the art and craft of maintaining high level political support for the principle, then the details, of HiAP.
9. *Have policy implementation barriers and facilitators been considered and integrated in policy formulation?* Note the need for policy clarity, organisational capacity, cross-sectoral support and partnerships, and to monitor and respond to limited progress.

## Discussion

Policy theories were not designed to support HiAP advocacy and programme evaluation. Using them instrumentally can be counterproductive if it introduces research design flaws. For example, core HiAP research questions relate to unfulfilled expectations: why is there such a gap between evidence and policy, expected and actual levels of joined-up government, or strategy and implementation? In contrast, the foundational question of policymaking research is: how do policy processes work? By asking it first, HiAP advocates could infuse their research designs with more realistic assumptions. To demonstrate, we describe the key elements of policy theories that combine to produce a narrative of policymaking. We apply this story to the HiAP’s narrative, playbook, and practical lessons.

## Three essential parts of a policy theory narrative

There are many policy theories serving different purposes. However, we would expect to find reference to three elements ( Cairney, 2020a: 229–34):

*1. The limits to policy change*

Empirical studies measure the speed and substance of policy change, in a context where we would expect to find minor change in most cases and major change in few. Studies describe the policy ‘tools’ or instruments to provide regulations, funding, and organisational or information-based support (2020a: 229; Hood & Margetts, 2007). Treating ‘policy’ as a large collection of instruments helps identify a range of commitments, from *maximal,* built on an ambitious formal strategy backed by regulations, redistributive tax and spending, and major government resources, to *minimal,* built on rhetoric and exhortation to individuals with no evidence of government investment ( Cairney & St Denny, 2020: 18). Either way, governments are modifying policies rather than making something new. A government’s initial commitment is a poor guide to outcomes, since it is difficult to predict how a new agenda interacts with existing commitments ( Cairney, 2020a: 229). We do not know the meaning of HiAP unless we relate it to policy as a whole.

*2. The limits to processing evidence*

Policy theories identify a key element of ‘bounded rationality’ ( Simon, 1976): individual policymakers (and organisations) can only process a tiny proportion of available information. The policymaking consequences can include: policymakers combine cognition and emotion (or adopt standard operating procedures) to prioritise some information and some issues and ignore the rest; they form coalitions with people who share their beliefs; they draw on emotion and simple stories to identify which populations deserve support or punishment; and, they rely on trial-and-error or instinct to navigate policy processes.

Consequently, the use of evidence to ‘frame’ issues or ‘learn’ how to understand and solve policy problems ‘is a political process in which actors engage selectively with information, not a rational search for truth’ ( Cairney, 2020a: 231; Dunlop & Radaelli, 2018). Policymakers use scientific evidence to reduce their *uncertainty* (a lack of information on problems or solutions), but politics is a competition to reduce *ambiguity* (a lack of agreement on how to define the problem and decide which solutions are feasible). Ambiguity does not end when HiAP actors ‘reframe’ policy problems in relation to systems rather than lifestyles; *they alone do not have the power to make this judgement*. Nor does it end when policymakers express a vague commitment to HiAP. This act does not indicate the extent to which HiAP is a priority or the trade-offs they will make between preventive and reactive policies ( Cairney *et al.*, 2021).

*3. The limits to policymaker control*

Policymakers operate within policymaking environments that constrain and facilitate action. Five concepts help describe its dynamics ( Heikkila & Cairney, 2018):

1. *Actors*. Many people and organisations make and influence policy across many levels and types of government. There is no single ‘centre’ of government driving HiAP reforms. There are many ‘centres’ or ‘venues’ for authoritative choice ( Cairney *et al.*, 2019).
2. *Institutions*. Each venue contains formal and informal rules. Some are written and understood widely. Others are unwritten and communicated non-verbally ( Ostrom, 2007). Implicit rules may contradict formal commitments. HiAP advocates may be fluent in some but struggle to understand others.
3. *Networks*. Each venue has its own relationships between policymakers and influencers. HiAP advocates may be central to some and excluded from others.
4. *Ideas*. Different venues entertain different ways to understand the world and its policy problems. Public health ideas are taken for granted in one venue but alien in another.
5. *Policy context (or conditions)*. Socioeconomic factors include geography, demography, social attitudes, and economic activity. Policymakers respond to context and events, and use proxy indicators of context to identify the technical and political feasibility of HiAP solutions.

Knowledge of these elements is crucial to knowledge of HiAP. It is tempting to conclude that HiAP fails because politicians do not exhibit ‘political will’. However, this argument ignores the fact that HiAP begins as little more than a formal commitment, taken forward by policymakers with a limited overview of its implications and low control of policymaking from the ‘centre’. They set strategic aims, but *delivery* across and outside of government depends on the behaviour of many actors in an environment over which no-one has full understanding. This connection of ‘political will’ to a wider context should be a routine feature of HiAP studies ( Baum *et al.*, 2020: 2).

## Implications for the HiAP narrative: revisit the logic of intersectoral action

*Public administration* research (on how to manage government and implement policy) informs and improves the HiAP agenda, while *public policy* research (explaining the dynamics of policy processes) challenges HiAP’s logic. 

***Studies of public administration enhance the evidence base for HiAP.*** Each aspect of HiAP intersectoral action – ‘joining up’ government, ‘boundary crossing’, and ‘partnership working’ - is researched extensively in public administration ( Carey & Friel, 2015: 796). Carey *et al.* (2014: 9) identify elements to foster a ‘supportive architecture’ for HiAP:

1. ‘Hard’ or ‘structural’ changes, including: a ‘mandate for change’ set by central government combined with sufficient control spread across multiple levels of government; clear ‘accountability and incentive mechanisms’; and sufficient resources, used flexibly in each relevant level of government.
2. ‘Soft’ or ‘cultural and institutional’ changes, including: a ‘strategic focus on collaboration’, ‘skill development’, a ‘rallying call’ for participants, and ‘information sharing’.

These elements come from Carey & Crammond’s (2015: 1022–8) review of the ‘joined-up government’ literature in which success depends on factors including:

- a ‘supportive architecture’, where agreed aims are matched to the means to achieve them, with enough flexibility to adapt to the dynamics of coordination efforts
- mutually reinforcing changes at multiple levels of government, reinforced by shared targets
- high commitment by politicians, to cut through ‘administrative silos’ and ‘turf wars’
- strong ‘leadership’ to ensure that all relevant bodies sign up to changes
- skilful actors, in problem-solving, coordination, brokering agreements, and engaging with non-governmental actors
- leaders able to work inside and outside formal arrangements (while respecting the link between action and accountability)
- a manageable number of aims and policy instruments
- a powerful narrative to challenge business-as-usual approaches and give people a common purpose.

Similarly, Greer & Lillvis (2014: 14–15) emphasise the role of ‘ *political leadership*’, including elected policymakers dedicated to producing detailed plans and targets, ‘ *bureaucratic change*’, such as forming or reorganising dedicated units, and creating new rules such as a requirement to use HIAs (and making rules hard to break), and ‘ *indirect strategies*’ to ‘permanently empower allies … to apply pressure and detect deviation’, such as giving data access to more organisations or establishing ombudsmen.

Some studies challenge HiAP assumptions or suggest modifications. For example, Carey *et al.* (2014: 9) argue that:

1. No single HiAP body can manage this process. HiAP is akin to the management of networks where any solution must be tailored to participants. The rigid centralisation of strategy will have unintended consequences.
2. There is no single HiAP model. All joined-up initiatives are tailored to participants.
3. HiAP-style reforms can be counterproductive and demoralising (see also Holt *et al.*, 2018).

As such, we can connect these insights to the wider literature to learn how to:

1. *Avoid inflated expectations for reforms*. ‘Decades of practical experimentation with public administration reform shows us that restructuring does not remove boundaries; it simply reconfigures them’ ( O’Flynn, 2016: 440).
2. *Avoid their unintended consequences.* Molenveld *et al.*’s review of policy coordination studies (2020: 9) identifies factors to aid motivation. Reforms should be: collaborative and not too hierarchical; tailored to local contexts rather than delivered uniformly; and, serious projects with clear aims backed by resources, because ‘some are disheartened by earlier government-wide approaches and want to see real action before they are willing to commit’.
3. *Identify how to foster cooperation*. Swann & Kim (2018: 273) identify the conditions conducive to collaboration among governing bodies, including: speaking directly and frequently with people, to help build ‘social capital’ and share information effectively; and, producing ‘small wins’ then working incrementally to reduce the burden of cooperation (2018: 286; see also Ansell & Gash, 2008).

Overall, we have reached a saturation point on practical advice for intersectoral action.

***Studies of policy processes help revisit HiAP assumptions and expectations.*** What we lack is a foundational discussion of the HiAP logic. In particular, policy theories provide reasons to manage expectations when we anticipate the impact of intersectoral action and collaboration.

First, classic studies of ‘policy communities’ highlight a logic of delegating policy responsibility to junior civil servants, engaged in routine consultation with a limited number of actors who trade information and advice for access ( Jordan & Cairney, 2013; Jordan & Maloney, 1997; Richardson & Jordan, 1979). Most policy is processed in silos that seem to defy central coordination. Silos develop rules appropriate to their own contexts, and their logics do not change simply because the overall effect looks like uncoordinated and incoherent policymaking ( Cairney *et al.*, 2020; Jordan & Halpin, 2006). The same is true of policymaking reforms, such as localism, that shift policy communities outside of central government departments ( Cairney & St Denny, 2020). While HiAP studies identify these dynamics as obstacles to be overcome, policy studies present them as ever-present forces to which to adapt. A lack of intersectoral action seems incoherent to some but *makes sense* to others.

Second, this contrast between overcoming versus adapting to policy dynamics is a general feature of ‘systems thinking’. Studies of policy analysis (employing a similar functional logic as HiAP) describe it as an effective strategy, in which ‘we can understand systems well enough to control, manage, or influence them’ ( Cairney, 2021a). In that context, Littlejohns & Wilson (2019) identify seven key elements, including ‘collaborative capacity’ and ‘leadership’. In contrast, studies of complex policymaking systems suggest that we reject the idea that policy outcomes are amenable to centralised coordination. Instead, outcomes ‘emerge’ from local interactions in complex systems, prompting us to adapt to the limits to central direction, such as: accepting that a policy that worked in one place or time may not work in another, giving more discretion to local actors to respond to their contexts, and engaging in trial-and-error policymaking to learn continuously from action, backed by realistic performance measures that do not punish alleged failure ( Cairney, 2020a: 107). If so, the idea of an *implementation gap* makes sense if viewed from the ‘centre’ of government, but has little meaning when viewed through the lens of complex systems thinking.

## Implications for the HiAP playbook: engage with governance dilemmas

Our discussion of the HiAP playbook invites researchers to take governance dilemmas seriously, focusing on the implications of collaboration and decentralisation for evaluation. Policymakers set aims that *seem* consistent in principle but are contradictory in practice. A key example is the use of evidence, which exposes the tension between two HiAP aims:

1. To encourage *centralisation*, to challenge health inequalities across whole populations, *formality*, to hold policymakers to account and ensure that governments deliver on their promises, and *uniformity*, to produce the ‘evidence base’ for the most effective HiAP interventions.
2. To encourage *decentralised* and *collaborative governance*, fostering cooperation with non-governmental actors and stakeholder ownership of policy, while avoiding ‘health imperialism’.

This dilemma is double-sided: trade-offs between governance aims interact with trade-offs when assigning value to knowledge ( Cairney, 2021b). In public health research, a key reference point is a ‘hierarchy’ of evidence quality based on research methods and publication in peer-reviewed scientific journals. It prioritises the systematic review of randomized control trials (RCTs), downplays the value of expert opinion, and does not feature experiential knowledge. Yet, HiAP is intersectoral. Advocates engage with many policy fields - including social work, social care, policing, and education – which signal more respect for service user, stakeholder, and practitioner experiences, often shared informally or in non-peer-reviewed publications. While these differences *seem* technical and resolvable by scientific principles, they are inescapably political ( Thomson, 2013; Cairney, 2021b, citing Althaus, 2020; Boaz *et al.*, 2019; Dobrow *et al.*, 2006; Fleming & Rhodes, 2018; Nutley *et al.*, 2013; Parkhurst, 2017; Petticrew & Roberts, 2006: 57–68; Smith, 2012; Stoker, 2010):

‘The former relates to the idea that we can generalize from common experience, while the latter emphasizes the complexity and uniqueness of each case and diversity of experience … Or, the latter relates explicitly to the politics of knowledge production and use, to criticise a narrow scientific view of evidence as exclusionary and contributing to the further marginalisation of vulnerable groups’ ( Cairney, 2021b).

Table 5 highlights these tensions by comparing two ideal-type models. Some governance and evidence choices seem to support each other. *Centralised governance* is conducive to the aim of rolling out uniform ‘evidence based’ interventions whose value is established in RCTs. A commitment to top-down policymaking, prioritising research methods, and fidelity to a ‘dosage’ precludes deliberation to share knowledge. *Collaborative governance* requires us to reject rigid claims to superior governing authority and scientific knowledge, focusing on deliberation and respect, generating ownership and trust via collective action ( Cairney, 2021b).

**Table 5.** Contrasting models of evidence-informed governance.

	Centralisation and ‘evidence-based policymaking' (EBPM)	Collaboration and story telling
The main story	EBPM requires a central government to roll out interventions based on the systematic review of randomised control trials.	Knowledge-informed policy requires collaboration across many ‘centres’, informed by participants telling stories of their policy relevant knowledge.
How should you gather evidence of effectiveness and best practice?	Using a hierarchy of evidence and methods.	Using principles of collaboration, knowledge sharing, and deliberation.
How should you ‘scale up’ from evidence of best practice?	Introduce the same model in each area. Require fidelity, to administer the correct dosage and measure its effect.	Tell stories based on your experience. Invite people to learn.
What should you prioritise?	The correct administration of the active ingredient.	Key principles, such as collaboration and respect for the knowledge of participants.

Source: adapted from ( Cairney, 2017; Cairney, 2020b; Cairney, 2021b) and Cairney & Oliver (2017).

It is tempting to address these dilemmas with bland pragmatism: let a thousand flowers bloom. However, it leaves two questions unresolved. First, do HiAP advocates ‘let go’ through *choice*, to reflect the benefits of methodological pluralism, or *necessity*, to adapt to their lack of control over these choices? Second, how would ‘letting go’ of the HiAP project help reduce health inequalities? HiAP programme logics seem to assume that collaboration can be treated instrumentally, as a way to deliver an outcome - health equity - identified in advance. However, a sincere commitment to collaborative governance suggests that this logic no longer applies.

## Implications for HiAP practical lessons: relate agency-centred stories to policymaking context

HiAP articles reinforce the idea that we can use insights from policy theories to support advocacy ( *policy theories for practical lessons*, above). Yet, their intended purpose is to produce scientific knowledge. While they provide an important way of thinking about policymaking, it is not obvious how they would translate into practical advice:

‘relatively abstract policy theories will rarely provide concrete advice of how to act and what to do in all given contexts. There are too many variables in play to make this happen. The complexity of policy processes, its continuously changing nature, and its diversity across contexts, prevent precise prediction for policy actors seeking influence or policy change’ ( Weible & Cairney, 2018: 186)
“If we simply connect lessons from theories to ‘what to do’ or how to influence a policy decision or outcomes, it disposes us to overextend our conclusions to contexts where they might not apply” ( Weible & Cairney, 2021: 202)

Theory-informed HiAP *advice* puts the agency of policy actors at centre stage (e.g. Khayatzadeh-Mahani *et al.*, 2018: 5–7). A small group of people draw lessons to influence policy: define the policy problem, learn how to manage networks and the ‘rules of the game’, show how contextual factors inform your predictions of your strategy’s impact and make informed action. Yet, policymaking *research* situates agency in a highly crowded and competitive political system: analysts face high uncertainty and ambiguity, there is contestation by many actors to define the policy problem, the rules of the game are unwritten, ill-understood, and not easily managed, the same strategy can succeed with one audience and fail with another, and windows of opportunity to secure policy change can be decades apart ( Cairney, 2021c).

Table 6 draws on the five key elements of policymaking environments to demonstrates the differences between these arguments, highlighting the ‘responses’ that may seem plausible to HiAP advocates and the ‘unresolved issues’ that warn against a simple translation exercise.

**Table 6.** the limits to agency-centred policy learning.

Issues	Responses	Unresolved issues
There are many policymakers and influencers spread across government	Identify the key venues for authoritative choices	It is difficult to know (a) from which venues to learn, and (b) which venues will seek to learn
Each venue has its own ‘institutions’ or the formal and informal rules	Learn the written/ unwritten rules of each relevant venue	Learning the rules is a long term and often infeasible process
Each venue has its own relationships between policy makers and influencers	Build trust and form alliances within networks	Trust formation is a lengthy commitment. Policymaking informality increases uncertainty about who is in charge
Each venue is guided by dominant ideas on the nature of problems and feasibility of solutions	Learn the language that actors use to frame problems and solutions	Dominant beliefs and language rule out many solutions as politically or technically infeasible
Policymaker attention is driven by changes in socioeconomic factors and events	Present solutions during periods of high attention to problems (‘windows of opportunity’)	HiAP actors do not cause the events that create windows of opportunity. They may be decades apart.

Source: adapted from Cairney (2021c)

In that context, we paraphrase van Eyk *et al.*’s (2019) study of healthy weight policy to accentuate this combination of optimism and caution ( Table 7).

**Table 7.** Practical HiAP advice with a dose of caution.

Advice	Qualification
Exploit a window of opportunity to ‘create acceptance’ for a HiAP approach to policy (backed by legislation or a ‘central mandate’)	Anticipate a ‘lack of sustained commitment’ particularly during changes to staffing and departments and budget cuts
Align the HiAP response to ‘existing mandates’ to create a ‘supportive authorising environment’ (win-win approach)	Expect existing mandates to prioritise economy over health frames, reducing public health budgets during state retrenchment.
Encourage actors to show leadership and become HiAP champions	Anticipate resistance if their message suggests ‘organisational culture change and changing established ways of operating’

Source: adapted from ( van Eyk *et al.*, 2019: 1169, Table 5)

This caution extends to the use of specific policy theories. First, avoid learning the wrong lessons. For example, we find sporadic use of the advocacy coalition framework (ACF), which describes: actors seeking to translate their beliefs into policy, forming coalitions with actors who share their beliefs, and competing with other coalitions in policy ‘subsystems’ ( Weible & Ingold, 2018). While it is possible to use the ACF to learn how to mobilise coalition allies ( Harris *et al.*, 2018), it would be missing the point to use ACF insights to *change the beliefs* of competitors ( Kongats *et al.*, 2019; Nykiforuk *et al.*, 2019).

Second, avoid exaggerating practical lessons. For example, the most popular theory is ‘multiple streams analysis’ (MSA) ( Cairney & Jones, 2016; Kingdon, 1984; Vassiliou *et al.*, 2020: 66). It describes a ‘window of opportunity’ for policy change when three streams’ come together:

1. Problem stream: there is heightened attention to a policy problem.
2. Policy stream: a technically and politically feasible solution is available.
3. Politics stream: policymakers have the motive and opportunity to select it.

Public health researchers seem drawn to MSA because it supports the HiAP playbook, highlighting ‘policy entrepreneurs’ with the resources to present their favoured solutions during a window of opportunity ( Cairney, 2018). Yet, MSA studies tell a different story, in which policymaking environments are the main source of explanation: generating often-infrequent and unpredictable opportunities, and constraining or facilitating entrepreneurial action (prompting most entrepreneurs to fail) ( Cairney, 2021a).

In that context, Kickbusch *et al.* (2014: 187–92; see also Baum *et al.*, 2015) provide a useful description of entrepreneur impact in South Australia, demonstrating that:

- *Entrepreneurial success is unusual*. Kickbusch and others were able to convince policymakers that a strategic focus on the social determinants of health across government could help reduce the unsustainable burden on health services. No other HiAP study describes this level of success.
- *HiAP success is to set the agenda, not deliver outcomes*. HiAP represented a way to influence the policymaking environment to make it more conducive to specific policy solutions. However, subsequent studies demonstrate its limited impact.

## Limitations

No search or review is comprehensive, and it is possible that a wider search process with (1) more keywords (e.g. ‘Healthy Cities’), and (2) the inclusion of books and policy documents would have yielded more insights (e.g. Clavier & de Leeuw, 2013; de Leeuw & Simos, 2017). However, a specific focus on HiAP and policymaking articles allowed us to (1) reach a saturation point, to make firm conclusions on a well-defined field, and (2) compare the results with studies in other sectors (beginning with Cairney & Kippin, 2021 on education equity). We perform wider searches for books and policy documents (based on techniques such as snowballing) in additional work (e.g. Cairney *et al.*, 2022). As discussed by Cairney & Kippin (2021), the more pressing limitation is a bias in research towards Global North experiences, exacerbated by our focus on English language articles. While the Results and Discussion sections identify key implications of limited HiAP progress, their global generalizability should not be assumed.

## Conclusions

We find a consistent and coherent HiAP narrative. It treats health as a human right and identifies health equity as its goal. Its social determinants lens helps quantify the problem of health inequalities and identify the evidence for upstream policy solutions. It promotes intersectoral and collaborative action, to recognise the lack of power of health actors to make the policies that improve population health and health equity. It seeks to generate and maintain political will, to set a radical new agenda and overcome obstacles to policy change.

This narrative informs a seven-point HiAP playbook. Use a well-established model to generate and maintain momentum. Raise awareness of HiAP and connect it to government business. Foster win-win solutions to build trust and ensure that HiAP is everyone’s business. Avoid the sense that HiAP symbolises ‘health imperialism’. Encourage policy champions. Use HiAP to improve the use of HIA. Reject narrow measures of HiAP’s value.

In practice, HiAP has proven to be a vague proposition backed by an ineffective playbook. Empirical studies encourage us to reject the sense that HiAP is a clearly defined model of policy and policymaking. They prompt us to take seriously the dilemmas that arise during attempts to implement HiAP. Country studies reinforce these conclusions. South Australia represents a best case example in which HiAP is embraced at a strategic level and delivered from the centre of government. Its experiences highlight high rhetorical commitment but few resources, a HiAP unit with low influence, and a tendency for HiAP to be overshadowed by ‘neoliberal’ policymaking, state retrenchment, and a commitment to protect reactive health services. Nordic countries represent best case examples of countries with a welfare state and a decentralised political system conducive to HiAP aims. Their experiences highlight a gulf between national commitment and formal action versus local variation and slow progress, and expose dilemmas in which the push for formal reforms and structural reorganisations undermines local collaborative action. Almost all country and comparative studies reinforce these general conclusions.

In other words, the dominant narrative of HiAP *in theory* does not correspond to the meaning of HiAP *in practice*. The former is an ambitious strategy to address the social determinants of health with radical policy change across multiple sectors, facilitated by intersectoral action and high strategic commitment to produce support for better policies. The latter is an ambitious strategy on paper only, representing moderate policy change at best and a negative commitment at worst, particularly when the funding and allocation of staff is minimal in relation to the wider sector. In that context of remarkably unequal resources and status, intersectoral action helps *dilute* the ambitions of HiAP enthusiasts, not produce policies they favour. HiAP’s meaning-in-practice relates to its low importance in relation to anti-HiAP elements, including limited economic redistribution, a small or reducing welfare state, high funding for healthcare, and performance and accountability measures that foster statutory duties, short term targets, and acute or reactive services.

HiAP studies draw on policy theories in that context. They seek practical lessons to improve the HiAP playbook, evaluation, and intersectoral and collaborative action. However, policy theories were not designed for this purpose, and their instrumental use exposes this problem. Studies of practical lessons for advocates exaggerate the role of agency and downplays context. Studies of evaluation help visualise complexity without showing how to identify causality or navigate the process. HiAP studies of intersectoral action could learn from public administration to improve coordination, but do not acknowledge the powerful rationale of silo working and the inevitable limits to central coordinative capacity.

In other words, although they often draw on policy theories, most HiAP studies still treat policymaking as a technical exercise, to find the right language to define the problem, the solutions that work, and the right model of intersectoral action and implementation. They too often ignore power and politics. Or, they criticise the impact of politics on policy while presenting functionalist arguments: identifying which policies *should* be selected, and how policymaking *should* work, rather than what actually happens. HiAP studies need to move from *assumptions* about the benefits of collaboration and policy instruments to *evidence* from case studies of implementation and evaluation. Until then, there is great potential to keep emphasising aspirational terms without examining their well-documented limitations.

Policy theories can help provide practical lessons, but to serve critical reflection rather than a HiAP playbook. They help explain an ‘implementation gap’ by showing that policy outcomes are beyond the control of policymakers and HiAP advocates. This explanation should prompt reflection on what ‘implementation gaps’ *mean*, and how to adapt to them, rather than simply how to close them. Similarly, they help explain an ‘evidence-policy’ gap. However, they highlight the role of political choice in evidence and governance dilemmas, and expose trade-offs between a desire for uniform outcomes (to produce health equity) and acceptance of major variations in HiAP policy and policymaking. While a core group knows and shares this perspective, we find that most HiAP scholars remain anchored to an unrealistic and unhelpful view of politics, power, and policymaking.

## Data availability

### Underlying data

All data underlying the results are available as part of the article and no additional source data are required.

### Extended data

Figshare: The future of public health policymaking after COVID-19: a qualitative systematic review of lessons from Health in All Policies

https://doi.org/10.6084/m9.figshare.14216774.v1

This project contains the following underlying data:

- Cairney St Denny Mitchell 2021 HiAP bibliography 12.3.21.pdf (A structured bibliography to accompany: The future of public health policymaking after COVID-19: a qualitative systematic review of lessons from Health in All Policies)

### Reporting guidelines

OSF: PRISMA checklist for ‘The future of public health policymaking after COVID-19: a qualitative systematic review of lessons from Health in All Policies’

https://doi.org/10.17605/OSF.IO/SEKBW

Data are available under the terms of the Creative Commons Zero “No rights reserved” data waiver (CC0 1.0 Public domain dedication).
