# Supplementary material for: The future of public health policymaking after COVID-19: a qualitative systematic review of lessons from Health in All Policies
Source: Open Res Eur. 2021 Jul 21;1:23. Originally published 2021 Mar 24. [Version 2] doi: 10.12688/openreseurope.13178.2 (PMC10445916; doi:10.12688/openreseurope.13178.2)
Supplement: Peer review history

# Peer review: Reviewer response for version 1

**DOI:** 10.21956/openreseurope.14248.r27101

**Published:** 2021-06-28

**Authors:** Charlotte Godziewski

**Affiliations:** 1 Sociology and Policy, School of Social Sciences and Humanities, Aston University, Birmingham, UK

This review presents an extremely detailed and thorough account of what ‘Health in All Policies’ (HiAP) is, and the issues faced by HiAP advocates, which are often thought of in terms of an ‘implementation gap’. This refers to a disappointing discrepancy between what HiAP is supposed to look like, and how it translates into real-life, concrete practice. The authors start by outlining the main points of what they see is a coherent yet vague HiAP narrative. They then explain how most HiAP advocates develop a strategy (playbook) for making this vision a reality. This strategy has common traits, but also a large range of updates and changes in response to identified challenges. However, the perceived ‘implementation gap’ remains.

 In the discussion, the authors explain what they think are some of the causes of these ‘unfulfilled expectations’ (p.23):

 Mainly, they claim that HiAP advocates’ tendency to instrumentally retrofit policy theories to their policy agenda is a problematic starting point. This leads to unrealistic expectations of policy change because the realities of the policymaking environment are not given enough consideration. Rather than focusing on understanding the policymaking environment, HiAP research keeps producing practical advice for intersectoral collaboration, which has now ‘reached a saturation point’ (p.24)

 The authors also highlight some inherent contradictions contained in HiAP which need to be engaged with, in particular with regards to centralization versus decentralization; top-down versus bottom-up; and evidence-based policymaking versus ‘grounded knowledge’. I believe this ultimately boils down to deeper philosophical tensions between ‘facts and norms’, technocratic legitimacy versus democratic legitimacy and so on. 

 I very much enjoyed reading this detailed and insightful review, it provides a very useful way to gain perspective and reflect on the frustrating question ‘why isn’t HiAP working?’. In my work, I have explored this question, yet instead of thinking about policy outcomes, I examined why HiAP means so many different things to different people, and through what processes those meanings change. I concur with the author’s suggestion that HiAP research needs to engage more seriously with policy theory as well as critical political economy.

Are sufficient details of the methods and analysis provided to allow replication by others?

Yes

Are the rationale for, and objectives of, the Systematic Review clearly stated?

Yes

Is the statistical analysis and its interpretation appropriate?

Yes

Are the conclusions drawn adequately supported by the results presented in the review?

Yes

Reviewer Expertise:

My research looks at the politics/political economy of health promotion, with a current focus on Health in All Policies in the EU.

I confirm that I have read this submission and believe that I have an appropriate level of expertise to confirm that it is of an acceptable scientific standard.

# Response

**Published:** 2021-07-19

**Authors:** Paul Cairney

**Affiliations:** University of Stirling, UK

We thank Dr Godziewski for this thoughtful and constructive peer review.

# Peer review: Reviewer response for version 1

**DOI:** 10.21956/openreseurope.14248.r26623

**Published:** 2021-04-26

**Authors:** Elisabeth Fosse

**Affiliations:** 1 Department of Health Promotion and Development, University of Bergen, Bergen, Norway

This is a very comprehensive, systematic literature review of research on Health in all policies (Hiap). It is not a systematic review in the original sense, and this means in this case that there is not a statistical analysis. The opportunity to review this type of review should be included in the criteria, I ticked the box for statistical analysis to be able to proceed with my review.

 I have been working in the field of Hiap for many years, and I find this literature review the most extensive, comprehensive and meaningful I have read so far. The authors cover research from most corners of the world, including non-western countries. They describe and analyse the contributions from the point of departure of what they call the “Hiap playbook” and are thus able to describe and critically analyse the main themes applied by scholars working with Hiap.

 Still, I find the most valuable contribution the critique presented regarding the underlying perceptions of, at least parts of the Hiap playbook. To a great extent, this builds on an instrumental view of public policy, where authors prescribe and suggest how to achieve Hiap, but often without understanding the complexity of policy and policy analysis. One important contribution of this review, is that it provides insight into the field of policy analysis and suggest how this could be applied to the analysis of policies, including important themes like health issues, intersectoral collaboration and health inequalities.

Are sufficient details of the methods and analysis provided to allow replication by others?

Yes

Are the rationale for, and objectives of, the Systematic Review clearly stated?

Yes

Is the statistical analysis and its interpretation appropriate?

Yes

Are the conclusions drawn adequately supported by the results presented in the review?

Yes

Reviewer Expertise:

Health promotion and public health policy analysis, in a Nordic and European context. Analysis of policies to reduce social inequalities in health. Research on Health in all policies and intersectoral collaboration.

I confirm that I have read this submission and believe that I have an appropriate level of expertise to confirm that it is of an acceptable scientific standard.

# Response

**Published:** 2021-04-27

**Authors:** Paul Cairney

**Affiliations:** University of Stirling, UK

We thank Professor Fosse for such a thoughtful and constructive peer review.
